# Electrokinetic and in situ spectroscopic investigations of CO electrochemical reduction on copper

**DOI:** 10.1038/s41467-021-23582-2

**Published:** 2021-06-01

**Authors:** Jing Li, Xiaoxia Chang, Haochen Zhang, Arnav S. Malkani, Mu-jeng Cheng, Bingjun Xu, Qi Lu

**Affiliations:** 1 State Key Laboratory of Chemical Engineering, Department of Chemical Engineering, Tsinghua University, Beijing, China; 2 Center for Catalytic Science and Technology, Department of Chemical and Biomolecular Engineering, University of Delaware, Newark, Delaware USA; 3 Department of Chemistry, National Cheng Kung University, Tainan, Taiwan; 4 College of Chemistry and Molecular Engineering, Peking University, Beijing, China

**Keywords:** Electrocatalysis, Electrocatalysis

## Abstract

Rigorous electrokinetic results are key to understanding the reaction mechanisms in the electrochemical CO reduction reaction (CORR), however, most reported results are compromised by the CO mass transport limitation. In this work, we determined mass transport-free CORR kinetics by employing a gas-diffusion type electrode and identified dependence of catalyst surface speciation on the electrolyte pH using in-situ surface enhanced vibrational spectroscopies. Based on the measured Tafel slopes and reaction orders, we demonstrate that the formation rates of C_2+_ products are most likely limited by the dimerization of CO adsorbate. CH_4_ production is limited by the CO hydrogenation step via a proton coupled electron transfer and a chemical hydrogenation step of CO by adsorbed hydrogen atom in weakly (7 < pH < 11) and strongly (pH > 11) alkaline electrolytes, respectively. Further, CH_4_ and C_2+_ products are likely formed on distinct types of active sites.

## Abstract (web summary)

Electrokinetic results are key to understanding the mechanisms in electrochemical CO reduction reaction. Here, the authors determine mass transport free kinetics using a gas-diffusion electrode and identified dependence of copper surface speciation on the electrolyte pH using in situ surface enhanced spectroscopies.

## Introduction

Electrochemical reduction of CO_2_ (CO_2_RR) into value-added chemical feedstocks and fuels offers a promising strategy to store the renewable electricity and close the carbon loop^1–4^. Copper-based electrocatalysts stand out in catalyzing CO_2_ electroreduction for their distinct capability to form hydrocarbons and oxygenates^5–8^. However, a number of fundamental questions remain unresolved, which pose challenges in further enhancing the rate and selectivity for valuable products, as well as improving the energy efficiency of the overall process for practical applications^9–11^. The extensive discussion of the impact of the electrolyte alkalinity on the CO_2_RR represents one such challenge that is of both fundamental and practical importance. On the practical front, a clear understanding could enable the use of less caustic electrolytes in the CO_2_RR, while maintaining the high rates and selectivity achieved in highly alkaline conditions by engineering the catalyst composition and/or the electrolyte^12^. The use of highly alkaline electrolytes inevitably leads to neutralization of the electrolyte with CO_2_, which is often unaccounted for in the energy efficiency analysis^13^. On the fundamental level, the electrolyte alkalinity could affect the electrode-mediated reaction by altering the thermodynamic driving force on a specific potential scale depending of the mechanistic details of different products, i.e., the standard hydrogen electrode (SHE) or the reversible hydrogen electrode (RHE). Recent discoveries of the pH dependence of Cu surface speciation at potentials relevant to the CO_2_RR raised the possibility that the active site or phase of the copper-based catalysts for all or some products in the reaction could be different in electrolytes with varying alkalinity^14^. Further, electrokinetic investigations at different pH could provide key insights into the CO_2_RR mechanism. Experimental mechanistic study of the CO_2_RR in aqueous electrolyte is challenging, because the multitude of chemical equilibria among CO_2_, hydroxide, bicarbonate, and carbonate make the isolation of roles of any specific species in the reaction difficult, which in many cases lead to contradictory conclusions^15–17^. In this regard, computational investigations have been informative in identifying the dominant reaction pathways towards hydrocarbons and oxygenates^18–26^. Despite significant research efforts and progress, there has been a lack of consensus regarding the key reaction steps forming multicarbon (C_2+_) products and CH_4_^18–25^. For C_2+_ product formations, it is widely accepted that the C–C coupling process is involved in the key steps of their reaction pathways^18–25,27–30^. Numerous reactions have proposed as potential rate-determining steps (RDSs) as follows: (1) dimerization of two surface-adsorbed *CO^18,20,22,25,27–29^; (2) dimerization of one surface-adsorbed *CO with one unabsorbed CO molecule (CO_b_)^30^; and (3) protonation of *CO to *CO(H) followed by coupling with *CO^21^ or *CO(H)^19^ to form the C–C bond. For CH_4_ formation, the RDS is considered to be the protonation of *CO to *CO(H)^18,19,23,31^. A summary of these proposed reaction schemes, as well as a number of other possible ones, is shown in Table 1.

**Table 1:** Summary of proposed reaction schemes for C_2+_ product (A.1–A.4) and CH_4_ (B.1–B.6) formation, and their corresponding Tafel slopes (detailed derivations are shown in Supplementary Note 1).

	Proposed reaction scheme for C_2+_ product formation	Tafel slope^a^	CO order at high *θ*_CO_	pH dependent
A.1	${{\,\!}^{\ast} {\rm{CO}}+{\,\!}^{\ast} {\rm{CO}}+{\rm{e}}}^{-}\mathop{\longrightarrow }\limits^{{\rm{RDS}}}{{\,\!}^{\ast} {\rm{C}}}_{2}{{{\rm{O}}}_{2}}^{-}+{\,\!}^{\ast}$	118 mV dec^−1^	0	No
A.2	${{\,\!}^{\ast} {\rm{CO}}+{\rm{CO}}}_{{\rm{b}}}{+{\rm{e}}}^{-}\mathop{\longrightarrow }\limits^{{\rm{RDS}}}{{\,\!}^{\ast} {\rm{C}}}_{2}{{{\rm{O}}}_{2}}^{-}$	118 mV dec^−1^	1	No
A.3	${{\,\!}^{\ast} {\rm{CO}}+{\rm{H}}}_{2}{{\rm{O}}+{\rm{e}}}^{-}\mathop{\longrightarrow }\limits^{{\rm{RDS}}}{{\,\!}^{\ast} {\rm{CO}}({\rm{H}})+{\rm{OH}}}^{-}$ ${{\,\!}^{\ast} {\rm{CO}}+{\,\!}^{\ast} {\rm{CO}}({\rm{H}})+{\rm{e}}}^{-}\to {{\rm{C}}}_{2}{{\rm{O}}}_{2}{({\rm{H}})}^{-}+{\,\!}^{\ast}$ or ${\,\!}^{\ast} {\rm{CO}}({\rm{H}})+{\,\!}^{\ast} {\rm{CO}}({\rm{H}})\to {{\,\!}^{\ast} {\rm{C}}}_{2}{{\rm{O}}}_{2}{({\rm{H}})}_{2}+{\,\!}^{\ast}$	118 mV dec^−1^	0	No
A.4	${{\,\!}^{\ast} {\rm{CO}}+{\rm{H}}}^{+}{+{\rm{e}}}^{-}\mathop{\longrightarrow }\limits^{{\rm{RDS}}}{\,\!}^{\ast} {\rm{CO}}({\rm{H}})$ ${{\,\!}^{\ast} {\rm{CO}}+{\,\!}^{\ast} {\rm{CO}}({\rm{H}})+{\rm{e}}}^{-}\to {{\rm{C}}}_{2}{{\rm{O}}}_{2}{({\rm{H}})}^{-}+{\,\!}^{\ast}$ or ${\,\!}^{\ast} {\rm{CO}}({\rm{H}})+{\,\!}^{\ast} {\rm{CO}}({\rm{H}})\to {{\,\!}^{\ast} {\rm{C}}}_{2}{{\rm{O}}}_{2}{({\rm{H}})}_{2}+{\,\!}^{\ast}$	118 mV dec^−1^	0	Yes
	CH_4_ formation			
B.1	${{\rm{H}}}_{2}{{\rm{O}}+{\rm{e}}}^{-}+{\,\!}^{\ast} \to {{\,\!}^{\ast} {\rm{H}}+{\rm{OH}}}^{-}$ ${\,\!}^{\ast} {\rm{CO}}+{\,\!}^{\ast} {\rm{H}}\mathop{\longrightarrow }\limits^{{\rm{RDS}}}{\,\!}^{\ast} {\rm{CO}}({\rm{H}})+{\,\!}^{\ast}$	59 mV dec^−1^	Negative	Yes
B.2	${{\rm{H}}}_{2}{{\rm{O}}+{\rm{e}}}^{-}+{\,\!}^{\ast} \mathop{\longrightarrow }\limits^{{\rm{RDS}}}{{\,\!}^{\ast} {\rm{H}}+{\rm{OH}}}^{-}$ ${\,\!}^{\ast} {\rm{CO}}+{\,\!}^{\ast} {\rm{H}}\to {\,\!}^{\ast} {\rm{CO}}({\rm{H}})+{\,\!}^{\ast}$	118 mV dec^−1^	Negative	No
B.3	${{\,\!}^{\ast} {\rm{CO}}+{\rm{H}}}_{2}{{\rm{O}}+{\rm{e}}}^{-}\mathop{\longrightarrow }\limits^{{\rm{RDS}}}{{\,\!}^{\ast} {\rm{CO}}({\rm{H}})+{\rm{OH}}}^{-}$ ${{\,\!}^{\ast} {\rm{CO}}({\rm{H}})+{\rm{H}}}_{2}{{\rm{O}}+{\rm{e}}}^{-}\to {{\,\!}^{\ast} {\rm{CO}}({\rm{H}})}_{2}{+{\rm{OH}}}^{-}$	118 mV dec^−1^	0	No
B.4	${{\,\!}^{\ast} {\rm{CO}}+{\rm{H}}}_{2}{{\rm{O}}+{\rm{e}}}^{-}\to {{\,\!}^{\ast} {\rm{CO}}({\rm{H}})+{\rm{OH}}}^{-}$ ${{\,\!}^{\ast} {\rm{CO}}({\rm{H}})+{\rm{H}}}_{2}{{\rm{O}}+{\rm{e}}}^{-}\mathop{\longrightarrow }\limits^{{\rm{RDS}}}{{\,\!}^{\ast} {\rm{CO}}({\rm{H}})}_{2}{+{\rm{OH}}}^{-}$	39 mV dec^−1^	0	yes
B.5	${{\,\!}^{\ast} {\rm{CO}}+{\rm{H}}}_{2}{{\rm{O}}+{\rm{e}}}^{-}\mathop{\longrightarrow }\limits^{{\rm{RDS}}}{{\,\!}^{\ast} {\rm{CO}}({\rm{H}})+{\rm{OH}}}^{-}$ ${\,\!}^{\ast} {\rm{CO}}({\rm{H}})+{\,\!}^{\ast} {\rm{H}}\to {{\,\!}^{\ast} {\rm{CO}}({\rm{H}})}_{2}+{\,\!}^{\ast}$	118 mV dec^−1^	0	No
B.6	${{\,\!}^{\ast} {\rm{CO}}+{\rm{H}}}_{2}{{\rm{O}}+{\rm{e}}}^{-}\to {{\,\!}^{\ast} {\rm{CO}}({\rm{H}})+{\rm{OH}}}^{-}$ ${\,\!}^{\ast} {\rm{CO}}({\rm{H}})+{\,\!}^{\ast} {\rm{H}}\mathop{\longrightarrow }\limits^{{\rm{RDS}}}{{\,\!}^{\ast} {\rm{CO}}({\rm{H}})}_{2}+\ast$^b^	59 mV dec^−1^	Negative	Yes

^a^Calculated assuming a symmetry factor of 0.5 in the RDS.

^b^The coverage of *H was typically considered to be small due to its weak hydrogen adsorption energy and insensitive to the applied potential.

The electrochemical CO reduction reaction (CORR) is an advantageous proxy in the mechanistic study of the CO_2_RR. It is known that CO is a necessary intermediate in the CO_2_RR and thus the two reactions share the pathways toward methane and C_2+_ products^22,23,28,32,33^. The neutral nature of CO alleviates the complexity of the multiple equilibria between CO_2_ and aqueous electrolytes of varying alkalinities. Meanwhile, the low solubility of CO (~1 mM) in aqueous electrolytes makes electrokinetic measurements susceptible to mass transport limitations^27,28^, especially at higher overpotentials, leading to nonlinear Tafel plots^32^. The convolution between electrokinetic and mass transport effect complicates quantitative microkinetic modeling^23^. Mass transport limitations could be partially alleviated by using flow-type reactors^12,34–36^; however, these configurations also introduce additional complexities to electrode/electrolyte interfaces and flow patterns for the benefit of high current densities, making them unsuitable for mechanistic investigations^37–40^.

In this work, we systematically determined Tafel slopes and CO reaction orders in forming C_2+_ products and CH_4_ at electrolyte pH from 7 to 14 using our recently developed polycrystalline Cu electrode with a gas-diffusion mechanism in a standard three-electrode H-cell (Supplementary Fig. 1)^28^. We show that rates of C_2+_ products are limited by the first electron transfer process and rates determined at different electrolyte pH essentially overlap at the SHE scale. Meanwhile, methane production rates determined in different electrolytes overlap in neither the SHE nor the RHE scale. Together with in situ surface-enhanced infrared and Raman spectroscopic results, we conclude that methane and C_2+_ products are produced on sites with distinct properties. Possible reaction pathways and associated RDS are discussed in the context of electrokinetic and spectroscopic results.

## Results

### Tafel analysis of C_2+_ products

The steady-state activity and selectivity of the CORR in the electrolyte pH range of 7.2–13.9 were determined at potentials between −0.5 and −1.2 V vs. RHE, i.e., approximately −1.3 and −1.6 V vs. SHE (Supplementary Fig. 2). This potential range is selected to drive sufficient but not excessive current densities to conduct reliable kinetic measurements. Current densities in all electrolysis remain stable, suggesting the absence of catalyst deactivation. Identical concentration of sodium cation (1 M) is maintained in all electrolytes employed in electrolysis studies, to avoid the cation effect in comparing the CORR activity: 0.3 M NaH_2_PO_4_ + 0.35 M Na_2_HPO_4_ (pH 7.2), 1.0 M NaHCO_3_ (pH 9.0), 0.5 M Na_2_CO_3_ (pH 11.3), 0.1 M NaOH + 0.9 M NaClO_4_ (pH 12.9), and 1.0 M NaOH (pH 13.9)^15,41,42^. We note that the cathodic electrolysis in near-neutral electrolytes could cause a rise of local pH near the electrode surface due to the production of OH^−^ as a byproduct in the CORR^43–46^. We show that the influence of local pH rise is insignificant in this work by employing an established diffusion–reaction model^43,45,46^ as detailed in Supplementary Note 2 and Supplementary Fig. 3.

**Fig. 1:**
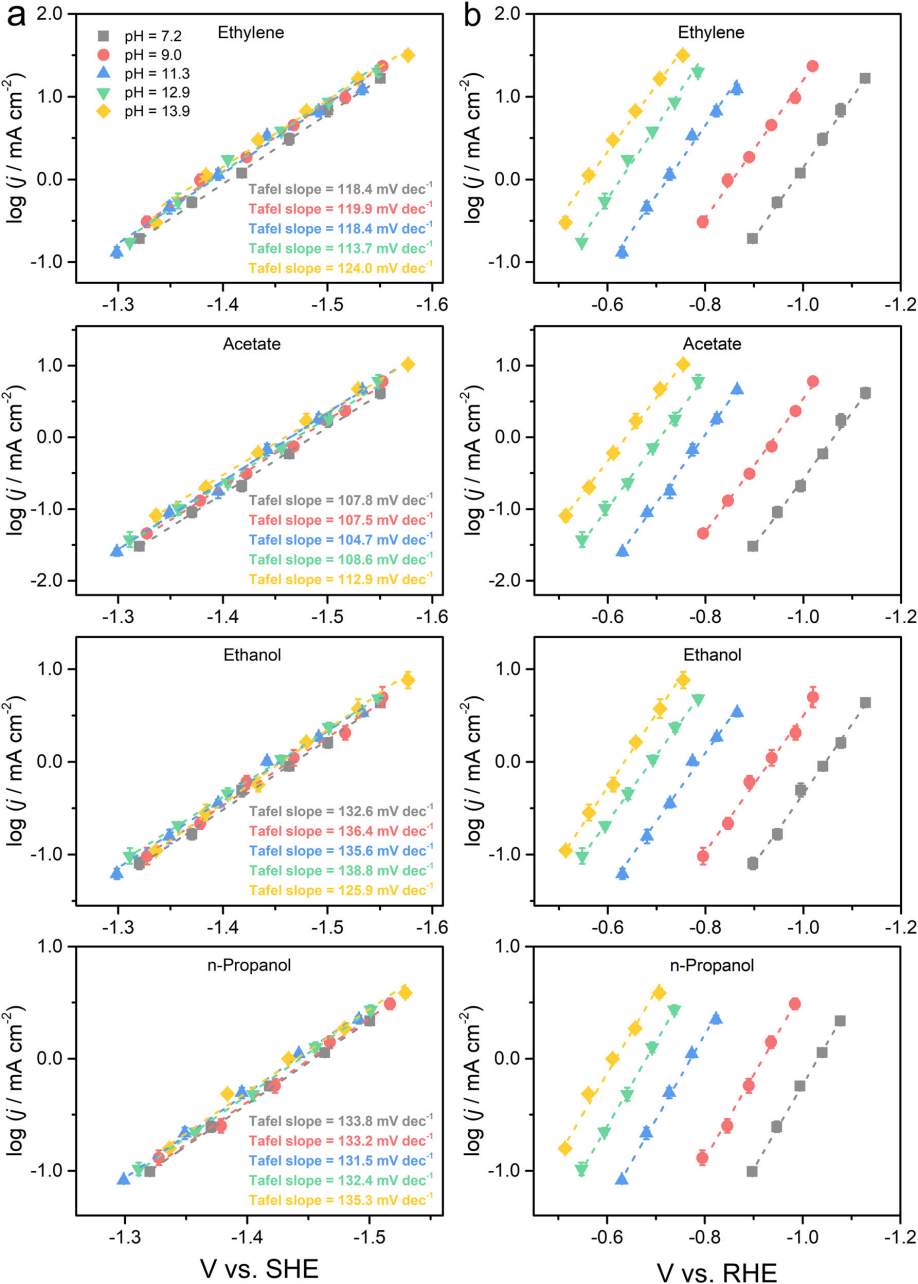
Tafel curves for C_2+_ product formation at different electrolyte pH. The logarithms of partial current densities for ethylene, acetate, ethanol, and *n*-propanol are plotted in SHE scale (**a**) and RHE scale (**b**), respectively. The error bars represent SD from at least three independent measurements.

Tafel slopes of all C_2+_ products were determined in a broad pH range of 7.2–13.9. Linear dependence of the logarithmic value of the partial current density (*j*) of C_2+_ products on the applied potential is observed (Fig. 1), suggesting the absence of mass transport limitation on the carbon-supported polycrystalline Cu dendritic microparticles (referred to as Cu MPs below). These results are in contrast with a previous report using Cu foils, on which clear mass transport limitation of CO was observed at more negative potentials^32,47^. The physical characterizations of Cu MPs were previously reported by our group^42^. All C_2+_ products exhibit a similar Tafel slope of ~118 mV dec^−1^ (Fig. 1), suggesting that their reaction kinetics is limited by the initial one-electron transfer process assuming a symmetry factor of 0.5. Thus, all RDS candidates that do not agree with this observation can be reasonably ruled out (a.1–a.3, Supplementary Table 1). Both CO dimerization (A.1 and A.2)^18,20,22,25,27–29^ and CO hydrogenation (A.3 and A.4)^19,21^ have been suggested by theoretical studies as potential RDS leading to C_2+_ products (Table 1). The list of potential RDS could be narrowed down by comparing the formation rates of C_2+_ products at different potential scales. If the RDS leading to C_2+_ products is *CO dimerization without involving a H^+^ transfer or a preceding proton transfer step, their rates are expected to be comparable on the SHE scale regardless of the electrolyte alkalinity. On the contrary, if the RDS or a preceding step involves a H^+^ transfer, e.g., CO hydrogenation with H^+^ (A.4), their rates should be similar on the RHE scale, to compensate for the difference in the H^+^ activity^32,41^. These deductions are based on the assumption that the electrolyte alkalinity does not impact the properties of active sites, which will be discussed in more detail later. Figure 1a, b show the Tafel plots of C_2+_ products on the SHE and RHE scales, respectively. Although the Tafel curves on the SHE scale overlap significantly over the entire electrolyte pH range investigated (Fig. 1a), they progressively shift to more positive potential on the RHE scale by ~ΔpH × 59 mV in more alkaline electrolytes (Fig. 1b). This result strongly indicates that the RDS of C_2+_ product formations does not involve a proton transfer or a preceding proton transfer step. Thus, CO dimerization via A.1 or A.2 and CO hydrogenation via A.3 are possible RDS candidates of RDS, and the SHE is the appropriate potential scale to compare the activities C_2+_ product formation evaluated in different electrolytes. This also confirms the validity of the assumption of the symmetry factor being 0.5. The RDS of C–C coupling involving a water molecule as the proton donor suggested by Liu et al.^23^ is kinetically indistinguishable from A.1, as in Eq. (1):

1 $$\ast {\rm{CO}}	+\ast {\rm{CO}}+{{\rm{H}}}_{2}{\rm{O}}+{{\rm{e}}}^{-}\leftrightarrow {[{\rm{OC}}\cdots {\rm{CO}}\cdots {{\rm{H}}}_{2}{{\rm{O}}}^{-}]}^{\dagger }\\ 	\to \ast {{\rm{C}}}_{2}{{\rm{O}}}_{2}{\rm{H}}+{{\rm{OH}}}^{-}$$

This is because the pH of the electrolyte impacts the free energy of neither the initial nor the transition state (TS). Similarly, although the net effect of the RDS in A.3 is to transfer a proton to adsorbed CO, the pH dependence is removed by considering H_2_O, rather than H^+^ or hydronium, as the proton donor in the RDS. The assumption of H_2_O as the sole or dominant proton donor is expected to hold only in alkaline electrolytes, in which the contribution of H^+^ as the proton donor is negligible due to its low concentration (activity). This condition is likely satisfied in the electrolytes employed in this work, as the lowest pH of the electrolyte is 7.2, in which the rate of reaction involving protons, as in Eq. (2):

2 $$\ast {\rm{CO}}+\ast {\rm{CO}}+{{\rm{H}}}^{+}+{{\rm{e}}}^{-}\to \ast {{\rm{C}}}_{2}{{\rm{O}}}_{2}{\rm{H}}+\ast $$

is expected to be insignificant due to the scarcity of H^+^^48^. Although buffer has been suggested as potential proton donors^41^, it does not appear to be the case in the current work due to the reasonable overlap of measured rates on the SHE scale in quite different electrolytes. We note that rates of C_2+_ products have a weak positive correlation with the electrolyte pH value (Fig. 1a and Supplementary Fig. 4), the potential reasons of which will be discussed below in the context of spectroscopic results.

A variation of Eq. (1), in which the C–C coupling and the hydrogenation of the resulting *OCCO intermediate occur sequentially, rather than in a concerted manner, is another possibility, as in Eqs. (3) and (4):

3 $$\ast {\rm{CO}}+\ast {\rm{CO}}\leftrightarrow \ast {\rm{OCCO}}+\ast $$

4 $$\ast {\rm{OCCO}}+{{\rm{H}}}_{2}{\rm{O}}+{{\rm{e}}}^{-}\leftrightarrow {[{\rm{OCCO}}\cdots {{\rm{H}}}_{2}{{\rm{O}}}^{-}]}^{\dagger }\to \ast {{\rm{C}}}_{2}{{\rm{O}}}_{2}{\rm{H}}+{{\rm{OH}}}^{-}$$

This mechanism regards the chemical C–C coupling (Eq. (3)) as a pseudo-equilibrated step. As no experimental observation of *OCCO exists, this mechanism necessarily entails that *OCCO is much higher in energy than adsorbed CO, leading to its low coverage. Meanwhile, the activation free energy of the C–C coupling must be significantly lower than that of the subsequent proton-coupled electron transfer (PCET, Eq. (4)) so that the C–C coupling is kinetically irrelevant. These energetic constraints make this pathway a less likely candidate than those discussed above.

### *p*_CO_ dependence of C_2+_ products

**Fig. 2:**
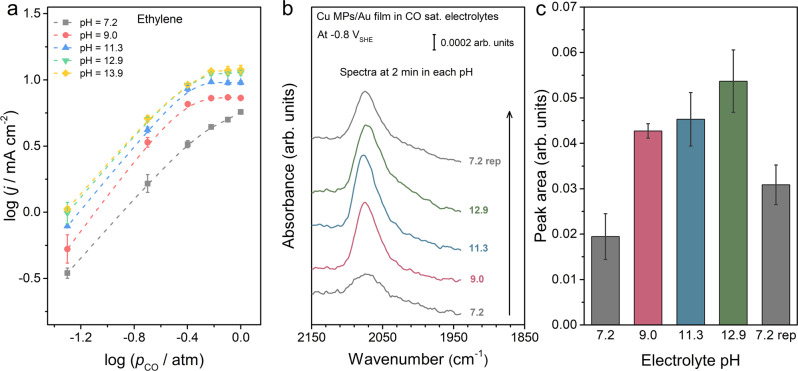
*p*_CO_-dependent C_2+_ product formation and spectroscopic studies at different electrolyte pH. **a** The logarithms of partial current densities for C_2_H_4_ formation vs. logarithms of *p*_CO_. The potential for all electrolysis is kept at −1.50 V vs. SHE. **b** SEIRA spectra of adsorbed CO on Cu MPs at −0.8 V vs. SHE in various electrolytes collected in a custom-designed spectroelectrochemical cell. **c** Integrated area of the CO adsorption peak in **b**. The error bars represent SD from at least three independent measurements.

Dependence of C_2+_ production rates on the CO partial pressure (*p*_CO_) was determined at −1.50 V vs. SHE with *p*_CO_ ranging from 0.05 to 1.0 atm. The dependence of formation rate on *p*_CO_ is similar for all C_2+_ products (Fig. 2a and Supplementary Fig. 5). In electrolytes with pH ≥ 9.0, rates of C_2+_ products increase with *p*_CO_ before plateauing as *p*_CO_ approaches 0.6 atm (Fig. 2a and Supplementary Fig. 5). This *p*_*CO*_ dependence makes A.2 unlikely, as the reaction order of CO should be no less than unity (derivation included in the Supplementary Note 1). Meanwhile, this type of *p*_CO_ dependence is quite characteristic of Langmuir–Hinshelwood kinetics (derivation included in the Supplementary Note 1), with the plateau at higher *p*_CO_ caused by a near saturation coverage of adsorbed CO. Assuming the Langmuir adsorption model, the value of the CO adsorption equilibrium constant (K_CO_^C2+^) is fitted to be 6.8–7.6 (Supplementary Table 2). We note that the absolute CO coverage of a saturation coverage is dependent on the composition and structure of the surface, as well as experimental conditions. The higher rates of C_2+_ products at 1 atm of CO is suggestive of higher absolute CO coverages. The lack of a plateau in the C_2+_ rates at *p*_CO_ ~1 atm in the electrolyte at a pH of 7.2 indicates a saturation coverage of CO, observed in the in more alkaline electrolytes, has yet been reached.

### In situ surface-enhanced vibrational spectroscopic investigations

The relative CO surface coverages in different electrolytes are estimated by surface-enhanced infrared absorption spectroscopy (SEIRAS). Spectra were collected on Cu MPs supported on a SEIRAS active Au film at −0.8 V vs. SHE, at which no CO band is expected on the underlying Au film^16^. All spectra were collected on the same catalyst in a custom-designed spectroelectrochemical cell by sequentially flowing different CO-saturated electrolytes to allow for reliable peak area comparison by avoiding film-to-film variations (Supplementary Fig. 6). No appreciable CORR occurs at −0.8 V vs. SHE, so the intensity of the CO band is representative of the its coverage in 1 atm CO in the respective electrolyte. A band at 2075 cm^−1^ attributable to CO adsorbed on atop sites of the Cu surface is present in the electrolyte with a pH of 7.2 at −0.8 V vs. SHE (Fig. 2b). The area of this peak more than doubled when the electrolyte pH increased to 9.0 and then gradually increased by ~25% as the electrolyte pH increased to 12.9 (Fig. 2b, c). Given the negligible peak position shift in electrolytes with pH of 7.2 and 9.0, the dynamical dipole coupling does not appear to play a significant role in the integrated peak area^49^. This is because dynamical dipole coupling typically favors the higher wavenumber band as the coverage of adsorbed species increases^49^. Thus, the integrated area of CO band in this work is representative of the relative surface coverages. The observation of higher CO coverage at higher electrolyte pH supports the hypothesis that the higher C_2+_ production rates in more alkaline electrolytes on the SHE scale is due to higher CO coverage. Moreover, the lineshape of the CO band remain relatively similar in the pH range of 9.0–11.3, but the band is significantly broadened at a pH of 12.9. This is an indication that the Cu surface becomes less homogeneous in the strongly alkaline electrolyte. In addition, when the electrolyte pH is decreased from 12.9 back to 7.2, the CO band remains broad and the peak area is higher (by ~55%) than the initial band collected in the electrolyte with a pH of 7.2 (Fig. 2c). This is likely due to the irreversible change in the surface composition and structure induced by the exposure to alkaline electrolytes, as demonstrated with Raman results below.

**Fig. 3:**
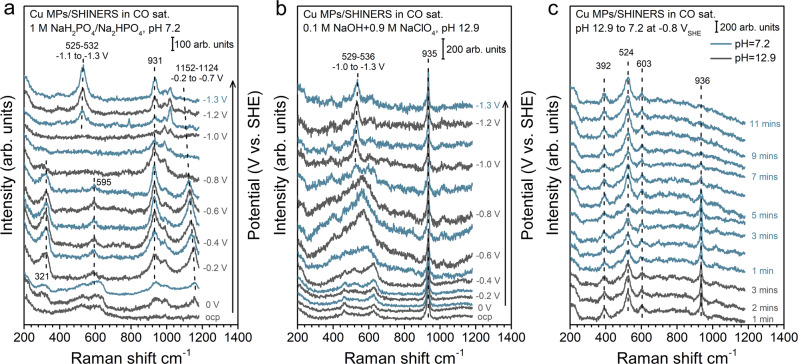
Potential-dependent SHINER spectra on Cu MPs at different electrolyte pH. SHINER spectra on Cu MPs in **a** 0.3 M NaH_2_PO_4_ + 0.35 M Na_2_HPO_4_ (pH 7.2); **b** 0.1 M NaOH + 0.9 M NaClO_4_ (pH 12.9) at potentials indicated in the figure. The spectra were collected at constant potentials with 0.1 V vs. SHE intervals in the cathodic direction from the OCP to −1.3 V vs. SHE and **c** SHINER spectra on Cu MPs at −0.8 V vs. SHE in the electrolytes switching from 12.9 to 7.2.

In situ shell-isolated nanoparticle-enhanced Raman (SHINER) spectroscopy in a custom-designed spectroelectrochemical cell is employed to probe the impact of the electrolyte on the surface speciation (Supplementary Fig. 7). Cu MPs do not exhibit strong surface enhancement of Raman signals and therefore silica-coated Au nanoparticles (Au@SiO_2_) are employed to enable the detection of interfacial signals^50,51^. At an electrolyte pH of 7.2, broad Raman bands at ~528 and 618 cm^−1^, attributable to surface Cu_2_O, appear at the open circuit potential, which is gradually reduced at below −0.2 V vs. SHE (Fig. 3a). A weak band centered at ~595 cm^−1^ is present at −0.2 to −0.7 V vs. SHE, which has been assigned to oxygen adatom on Cu (Cu-O_ad_)^14,51,52^. At potentials below −1.1 V vs. SHE, a well-defined band at ~530 cm^−1^ shows up and shifts to lower wavenumber as potential becomes more negative. This band has been assigned to a CuO_*x*_/(OH)_*y*_ mixed phase^14,51,52^. Importantly, at potentials below −0.2 V vs. SHE, bands corresponding to phosphates at 931 and 1152 cm^−1^ appear. The band at 1152 cm^−1^ shifts to lower wavenumbers as potential becomes more negative, whereas the 930 cm^−1^ band shifts more slowly in the opposite direction^53^. These potential dependent behaviors, referred to as the Stark tuning effect, indicate phosphate specifically adsorbs on the surface. A few bands in the 980–1022 cm^−1^ range also attributable to adsorbed phosphate appear at potentials below −0.8 V vs. SHE. No reliable Raman spectrum could be obtained at potentials below −1.3 V vs. SHE due to excessive bubble formation at high current densities. Raman band (~530 cm^−1^) corresponding to CuO_*x*_/(OH)_*y*_ shows up at increasingly less negative potential as the electrolyte becomes more alkaline (Fig. 3a, b and Supplementary Fig. 8), e.g., at −0.9 V vs. SHE in the electrolyte with a pH of 12.9 (Fig. 3b). This is in line with our recent pH dependence study of Cu surface speciation^14^. No other band attributable to adsorbed anions is observed in any other electrolyte investigated in this work, indicating the lack of specific adsorption. Only bands corresponding to anions in the bulk electrolytes are detected, e.g., 935 cm^−1^ for ClO_4_^−^ (Fig. 3b). Thus, the lower CO coverage in the phosphate electrolyte (pH 7.2) determined by SEIRAS is likely due to competitive adsorption of CO and phosphate. It is important to note that the impact of alkaline electrolyte on the surface speciation of Cu is irreversible. The CuO_*x*_/(OH)_*y*_ appears when Cu MPs are exposed to an electrolyte with a pH of 12.9 and persists when the electrolyte pH is decreased to 7.2 (Fig. 3c). This is consistent with the broader width and higher integrated area of the CO band in the electrolyte with a pH of 7.2 after the surface is exposed to a more alkaline environment, as compared to those on the fresh Cu MPs surface in the same electrolyte (top and bottom traces of Fig. 2b, respectively). It is a clear indication that the Cu surface speciation has a substantial impact on the coverage and heterogeneity of the adsorbed CO, which could be a cause to the slight increase in the production of C_2+_ products at higher electrolyte pH on the SHE scale (Fig. 1a).

### Tafel analysis and *p*_CO_ dependence of CH_4_ formation

**Fig. 4:**
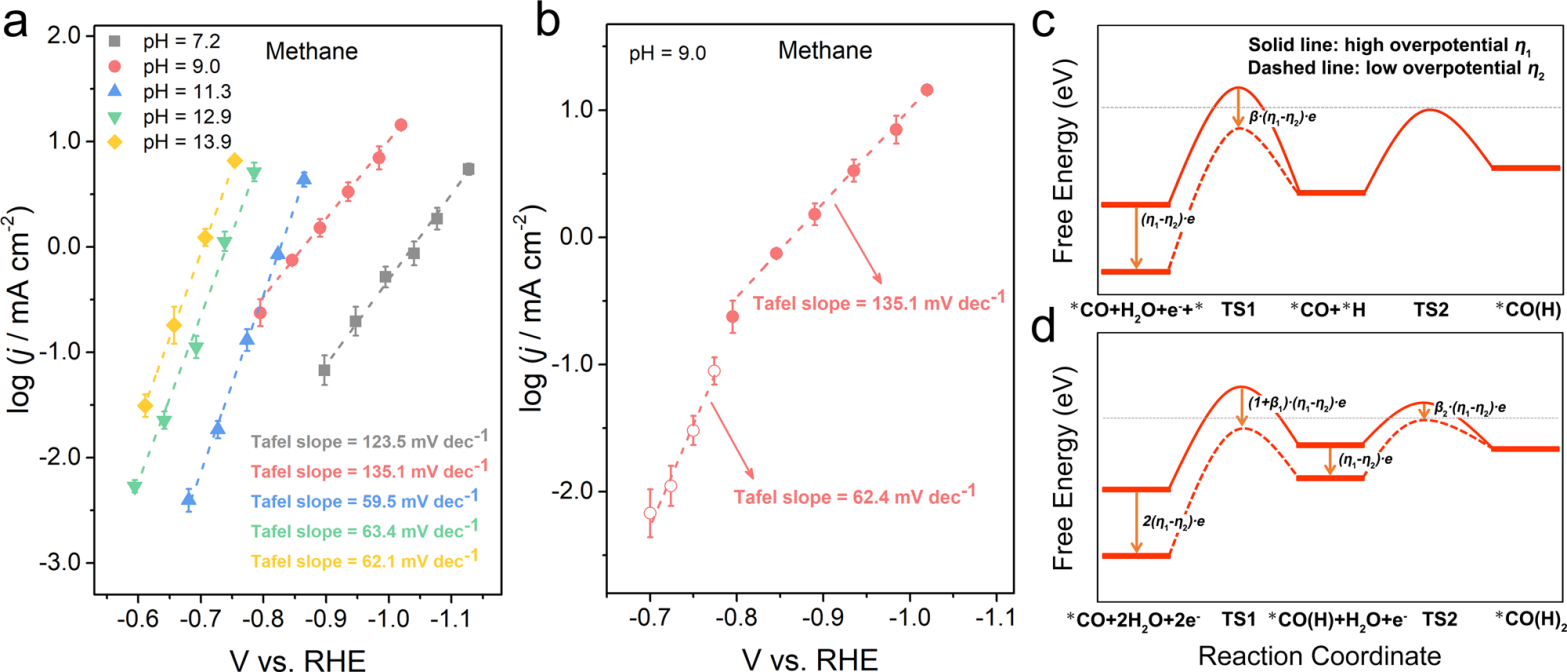
Tafel curves for CH_4_ formation at different electrolyte pH. **a** The logarithms of partial current densities for CH_4_ formation plotted in RHE scale. **b** The change of Tafel slope for CH_4_ formation at less biased potentials in electrolyte with pH 9.0. **c**, **d** Possible RDS shift of CH_4_ formation by decreasing overpotential. The error bars represent SD from at least three independent measurements.

**Fig. 5:**
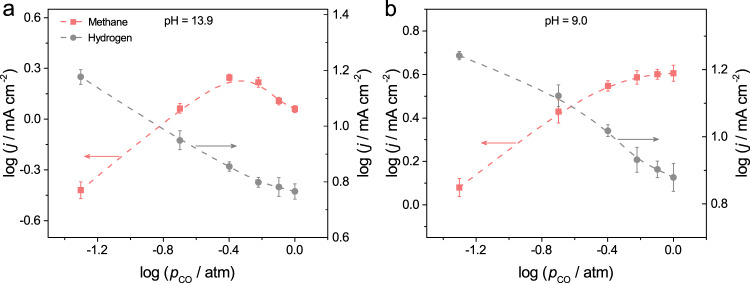
*p*_CO_-dependent CH_4_ formation rate at different electrolyte pH. The logarithms of partial current densities for CH_4_ formation vs. logarithms of *p*_CO_ at electrolyte pH of **a** 13.9 and **b** 9.0. The potential for all electrolysis is kept at −1.50 V vs. SHE (i.e., −0.68 V vs. RHE at pH 13.9 and −0.97 V vs. RHE at pH 9.0). The error bars represent SD from at least three independent measurements.

The nature and pH dependence of the RDS of CH_4_ formation is less clear. It is generally believed that one of the hydrogenation steps of CO is the RDS in the CH_4_ formation^18,19,22,27,28,31^. As in the kinetic analysis of C_2+_ products, we consider H_2_O, rather than H^+^, as the proton source due to the scarcity of H^+^ in neutral to alkaline electrolytes^48^. A few studies suggested that adsorbed *H from Volmer step could be a source of H (B.1 mechanism)^27,28^. For the sake of discussion, we plot the CH_4_ formation rate in different electrolytes on the RHE scale (Fig. 4a) and the same data plotted on the SHE scale are included in the Supplementary Information (Supplementary Fig. 9). The following three surprising observations are worth discussing: (1) unlike in the case of C_2+_ products, CH_4_ formation rates are dependent on the pH value of the electrolyte on both the RHE and the SHE scales; (2) the Tafel slope of methane formation is determined to be ~59 mV dec^−1^ in electrolyte with pH values above 11, but ~118 mV dec^−1^ in less alkaline electrolytes; and (3) in the electrolyte with a pH value of 9.0, the Tafel slope for CH_4_ formation switches from ~118 mV dec^−1^ at more negative potentials to ~59 mV dec^−1^ at > −0.8 V vs. RHE (Fig. 4b). Measurements cannot be reliably conducted in the less alkaline electrolyte (pH 7.2) at potentials > −0.8 V vs. RHE due to low current densities. Further, the dependence of the methane production rate on the CO partial pressure also varies with the electrolyte pH. Although the methane formation rate peaks at a *p*_CO_ of 0.4 atm in electrolytes with pH > 11, it increases monotonically with *p*_CO_ from 0 to 1 atm in less alkaline electrolytes (leveling off close to 1 atm, Fig. 5 and Supplementary Fig. 10). Below we discuss a few mechanistic hypotheses based on these experimental observations.

The electrolyte pH dependence of CH_4_ formation on the RHE scale is an indication that electrolytes impact the methane formation beyond the change in the proton activity. One possibility is the impact of different anions on the reaction, as demonstrated by Resasco et al^41^. We consider this unlikely to be the main cause in this work for two reasons as follows: (1) aside from the electrolyte with the lowest pH (7.2), no spectroscopic evidence of anion-specific adsorption is observed; and (2) the comparable C_2+_ production rates on the SHE scale in different electrolytes suggests that specific interaction between the anions and the surface intermediates is unlikely.

We first consider the mechanistic pathway in which the Volmer step is followed by a chemical hydrogenation step of CO. The Volmer step is generally believed to be the RDS for the hydrogen evolution reaction (HER)^54,55^. Assuming a symmetry factor of 0.5, a Tafel slope of ~118 mV dec^−1^ in electrolyte with pH values of 7.2 and 9.0 (≤ −0.8 V vs. RHE) suggests the first electron transfer as the RDS in the methane formation. The Volmer step could be the RDS in less alkaline electrolytes (B.2 mechanism). As reported previously^41^, the HER on Cu, as well as the RDS (the Volmer step), is accelerated in more alkaline electrolytes on the RHE scale, which is consistent with the hydrogen formation rate in our CO electrolysis studies (Supplementary Fig. 11a). Although the exact mechanism remains a topic of discussion, possible explanations include the presence of oxygen-containing species, which has been suggested to facilitate the H_2_O dissociation^48,56^ and/or the strengthening of the hydrogen-binding energy in more alkaline electrolytes^44,54^. The switch of the Tafel slope to ~59 mV dec^−1^ in electrolytes with pH values at or higher than 11.3 can be interpreted as a change in the RDS from the improved Volmer step to the subsequent chemical hydrogenation step (B.1 mechanism). The increase in the CH_4_ formation rate with the increase of the electrolyte pH can be rationalized with the improved Volmer step (pH 7.2 and 9.0) and the resulting higher *H coverage for hydrogenation (pH 11.3–13.9). We note that the absolute *H coverage on Cu likely remains low due to the lack of experimental observation and relatively low hydrogen-binding energy^54^. This reaction scheme can also rationalize the different Tafel slopes in the electrolyte with pH 9 at potentials below and above −0.8 V vs. RHE (Fig. 4b). By decreasing the applied overpotential, the free energy of the TS of the initial Volmer step would become lower relative to that in the subsequent chemical step as illustrated in Fig. 4c^57^. It follows that the RDS could be switched from the Volmer step to the subsequent hydrogenation process, which causes a shift of the corresponding Tafel slope from ~118 to ~59 mV dec^−1^ (from B.2 to B.1 mechanism). Further, a simple Langmuir–Hinshelwood model allows the mechanism B.1 to explain the observed *p*_CO_ dependence in more alkaline electrolytes. At high electrolyte pH, the chemical hydrogenation step in the B.1 mechanism is the RDS, which entails a site competition among *CO, *H, and unoccupied sites, which is typical in the Langmuir–Hinshelwood kinetics (Supplementary Note 1). Qualitatively, as the *CO approaches saturation, the methane formation becomes increasingly limited by the availability of *H, which is assumed to be in pseudo-equilibrium with unoccupied sites. This leads to a negative *p*_CO_ dependence at ≥0.6 atm, with a concomitant decline in the HER rate (Fig. 5a).

A few additional aspects of B.1 and B.2 mechanisms deserve further considerations. The B.2 mechanism entails that the methane formation and the HER share the same RDS, and in turn the same Tafel slope. Although the Tafel slopes for the HER in these electrolytes are significantly larger (>230 mV dec^−1^, Supplementary Fig. 11), it does not necessarily contradict this hypothesis because of the catalyst structure. We demonstrated in a recent work that the ability of the carbon-supported Cu MP catalysts to sustain much higher current densities in the CORR than that on Cu foils is primarily derived from the presence of the triple phase boundary^28^. Such triple phase boundary is not needed in the HER, as there is no gas-phase reactant. It follows that the HER occurs on both Cu sites at and away from triple phase boundaries (carbon support was shown to be largely inactive for the HER)^28,58,59^, whereas the CORR proceeds primarily on the former. As these two types of Cu sites are expected to have different CO coverages due to different CO mass transport capabilities to these sites, the HER could proceed via different mechanisms, leading to different Tafel slopes. Thus, the different Tafel slopes of the methane formation and the HER cannot be taken as definitive evidence against the hypothesis of the Volmer step as the RDS. A more serious flaw in the interpretation involving B.2 and B.1 mechanisms is the attribution of the shift the Tafel slope from ~118 to ~59 mV dec^−1^, to the change of the RDS from the Volmer step to the chemical step. This entails the assumption of the pseudo-equilibrium of the Volmer step in the B.1 mechanism, which contradicts the assumption that the Volmer step is the RDS in the HER. One elementary step cannot be simultaneously pseudo-equilibrated in one pathway, while the RDS in another, in a connected reaction network. Moreover, in electrolytes with lower pH values (9.0 and 7.2), the RDS of CH_4_ formation according to this rationale is the Volmer step (B.2 mechanism). A negative reaction order of CO (as in higher electrolyte pH) can be derived due to the existence of site competition between *H and *CO (Supplementary Note 1). However, no such negative reaction order is observed for CO in these electrolytes.

A second possible mechanism involves exclusively PCET steps in the hydrogenation of CO, as suggested by a recent computational study^23^. An electrochemical hydrogenation step of CO via the PCET (B.3 mechanism) as the RDS also agrees with the measured Tafel slope (~118 mV dec^−1^) at pH of 7.2 and 9.0 (≤ −0.8 V vs. RHE). The RDS is shifted to a later PCET step as the overpotential decreases or the electrolyte pH increases (B.4 mechanism), which would entail the symmetry factor of the RDS must significantly deviate from the commonly assumed value of 0.5 given the measured Tafel slopes of ~63 mV dec^−1^. This should not be considered as evidence against this hypothesis, because the value of the symmetry factor is dependent on the nature of the reaction rather than a fixed value. Similarly, it should also come as no surprise that the values of symmetry factors of the C_1_ and C_2+_ pathways could be different given their different pathways. The shift of the RDS to later elemental steps in an electrocatalytic pathway as overpotential decreases (as in the case of pH 9.0) is quite general^57^, as the driving force for later electrochemical steps attenuates more quickly than that of the earlier steps, as indicated in Fig. 4d. Meanwhile, the shift between B.3 and B.4 mechanisms, as in the case of B.1 and B.2 mechanisms discussed above, has trouble accounting for the observed *p*_CO_ dependence. Both mechanisms predict a monotonic rise of methane formation rate with *p*_CO_ (Supplementary Note 1), which is incompatible with the observed negative *p*_CO_ dependence in more alkaline electrolytes (Fig. 5a and Supplementary Fig. S10b, c).

The third mechanistic possibility we consider involves a PCET step in the initial CO hydrogenation step, followed by a chemical hydrogenation step. The PCET step is proposed to be the RDS in less alkaline electrolytes (pH 7.2 and 9.0), which is expected to yield a Tafel slope of ~118 mV dec^−1^ (B.5 mechanism). The RDS is shifted to the chemical step in more alkaline electrolytes, leading to a predicted ~59 mV dec^−1^ Tafel slope (B.6 mechanism). The shift in the Tafel slope with overpotential in pH 9.0 can be rationalized by a similar manner as discussed above (Fig. 4c). This framework also satisfactorily explains the observed *p*_CO_ dependence. When the PCET step is rate determining, a monotonic increase in the methane formation rate with *p*_CO_ is expected, which would gradually level off as the surface approaches the saturation CO coverage (Fig. 5b and Supplementary Fig. 10a). In contrast, site competition between *CO and *H is expected when the chemical hydrogenation step is the RDS, leading to a volcano shape *p*_CO_ dependence curve as observed in Fig. 5a and Supplementary Fig. 10b, c. Thus, the framework involving B.5 and B.6 mechanisms is consistent with all experimental observations reported in this work.

### Active sites for methane and C_2+_ products

The pH dependence of the methane production rate remains unresolved with the mechanistic discussion above and could afford insights into the identity of active sites for methane and C_2+_ products. Mechanisms for methane production discussed above predict that the rate should be comparable either on the SHE scale (B.2, B.3, and B.5 mechanisms) or on the RHE scale (B.1, B.4, and B.6 mechanisms), which is in disagreement with the reactivity results (Fig. 4a and Supplementary Fig. 9). Meanwhile, rates of C_2+_ products in different electrolytes largely overlap on the SHE scale, as predicted by the A.1 or A.3 mechanism. One potential explanation of this discrepancy is that methane and C_2+_ products are formed on two distinct types of active sites. Although the sites for C_2+_ products are relatively insensitive to the change in the surface speciation with the electrolyte pH, sites for methane production are more significantly impacted. The relative indifference of C_2+_-producing sites to the electrolyte pH, including the associated change in the surface speciation, makes the C_2+_ pathway proceed unaltered in different electrolytes. This hypothesis is consistent with our recent works showing that there is no direct correlation between the presence of the oxygen-containing Cu species on the surface and C–C coupling chemistry^14,51^. In contrast, properties of methane-producing sites appear substantially different in different electrolytes. The partial current density of C_2+_ products at 1 atm of CO in the electrolyte with a pH of 7.2 is ~20% lower than that at a pH of 9.0 at the same SHE potential. According to the A.1 mechanism, the reaction rate has a second-order dependence on CO coverage, which leads to an estimated ~11% lower CO coverage in the less alkaline electrolyte. A similar analysis for methane production rates (with B.5 mechanism) in these two electrolytes in the potential region on the SHE scale (where a Tafel slope of ~118 mV dec^−1^ was determined) shows an estimated 66% lower CO coverage in the less alkaline electrolyte with a first-order dependence on the CO coverage. The contrast between these estimates suggests sites for C_2+_ products and methane respond to electrolyte differently, and thus cannot be the same sites. We note that estimating the relative CO coverages on methane-producing sites based on methane production rates implicitly assumes the same rate constant in different electrolytes at the same SHE potential. Although this assumption is unlikely to hold rigorously because the CO-binding energy likely correlates with the methane formation activity, the conclusion of methane-producing sites are susceptible to the change in the electrolyte pH remains valid. A similar analysis according to the B.6 mechanism on the RHE scale in more alkaline electrolytes could be conducted with a similar conclusion. More generally, we can estimate the CO adsorption equilibrium constants (*K*_CO_) by fitting the rate expression with C_2+_ and methane formation rates in different electrolytes based on the proposed mechanisms discussed above. The *K*_CO_ values are consistently different when fitted with C_2+_ and methane formation rate data (Supplementary Table 2), which provides additional evidence, suggesting C_2+_ and methane are produced on sites with considerably different CO-binding energies. Based on the proceeding analyses and the Raman results, we propose that methane production enhanced on sites are either located close to the oxygen-containing surface Cu species, where the Cu surface structure is impacted by these neighboring species. Further, these methane-producing sites are largely ineffective in catalyzing the C–C coupling pathway. The understanding that methane and C_2+_ products are formed on different types of sites affords the prospect of developing catalysts selective for either products via site engineering.

## Discussion

In summary, we determined mass transport-free CORR kinetics, in a standard H-type electrochemical cell with a three-electrode setup, by employing a gas-diffusion-type polycrystalline copper powder electrode and identified dependence of catalyst surface speciation on the electrolyte pH using in situ surface-enhanced vibrational spectroscopies. Through combined electrokinetic and in situ spectroscopic investigations, we provide compelling experimental evidence that the formation rate of C_2+_ products in the CORR on Cu does not depend on the electrolyte pH and is limited by the first electron transfer without involving a proton. Although the C–C coupling step remains likely, the RDS in the formation of C_2+_ products, the possibility of hydrogenation of CO with water as the proton donor, cannot be ruled out. In contrast to C_2+_, methane production rates depend on the electrolyte pH in both the SHE and RHE scales. Methane production is limited by the CO hydrogenation step via a PCET in near-neutral electrolytes (7 < pH < 11) and a chemical hydrogenation step of CO by adsorbed hydrogen atom in more alkaline electrolytes (pH > 11). The different pH-dependent behaviors in formation rates of C_2+_ and methane, together with in situ surface-enhanced spectroscopic results, indicate that these two types of products are formed on distinct types of active sites.

## Methods

### Materials

Cu powder (<45 μm, 99.7% trace metals basis), sodium hydroxide (semiconductor grade, 99.9% trace metals basis), sodium carbonate (99.99% trace metals basis), phosphoric acid (trace metals basis), Chelex 100 sodium form, isopropanol (99.999% trace metal basis), and Nafion solution (5 wt %) were purchased from Sigma-Aldrich. Carbon monoxide (99.999%) and argon (99.999%) were purchased from Air Liquide. The carbon fiber paper support (Sigracet 39 BC) was purchased from the Fuel Cell Store. The electrolyte solutions were prepared using Milli-Q water (18.2 MΩ cm).

### Preparation of electrolytes

The sodium cation concentrations of all electrolytes were kept to be 1.0 M. The electrolyte pH was determined using an Orion Star™ A111 Benchtop pH Meter (Thermo Fisher Scientific). The electrolytes with the pH value of 13.9, 12.9, and 11.3 were prepared by dissolving 1.0 M NaOH, 0.1 M NaOH + 0.9 M NaClO_4_, and 0.5 M Na_2_CO_3_ in Milli-Q water (18.2 MΩ cm). To prepare the buffer electrolyte solutions with lower pH, 0.5 M Na_2_CO_3_ solution was first purged with CO_2_ gas (99.999%) for 12 h to obtain 1 M NaHCO_3_ solution. This electrolyte was then purged using Ar gas (99.999%) for an additional 5 h to remove the residue CO_2_ and achieve a pH value of 9.0. The electrolyte solution with pH 7.2 was prepared by dissolving 1.0 M NaOH and 0.65 M H_3_PO_4_ in Milli-Q water. All electrolytes were purified with Chelex 100 Resin prior to electrolysis.

### Preparation of polycrystalline Cu powder electrodes

To prepare the polycrystalline Cu MPs electrode, an ink solution was first prepared by mixing 8 mg Cu powder and 2.5 mL isopropanol followed by sonicating for 20 min. The ink solution was dropcasted onto a gas-diffusion layer of Sigracet 39 BC until a catalyst loading of 1 mg cm^−2^ was achieved. Next, 200 μL of a 2.5 wt% Nafion solution was uniformly deposited onto the catalyst layer. After drying at the ambient condition, the catalyst was further dried under vacuum to thoroughly remove the residual solvent. Then, the catalyst was cut into individual electrodes that were ~0.5 × 1.5 cm^2^. A nickel wire was attached using silver epoxy as the current collector.

### Electrochemical measurements

Electrochemical measurements were performed in an H-type electrochemical cell made from polymethyl methacrylate, to avoid possible Si contaminations. A piece of anion-conducting membrane (Selemion AMV AGC, Inc.) was used as the compartment separator. A graphite rod (Sigma-Aldrich, 99.999%) was used as the counter electrode and a Hg/Hg_2_Cl_2_ reference (saturated KCl, ALS Co., Ltd) was used as the reference electrode. The reference electrode was calibrated using a homemade SHE. The measured potential was converted to the RHE reference scale using the formulas E (vs. RHE) = E (vs. Hg/Hg_2_Cl_2_) + 0.241 V + 0.0591 V × pH. A Gamry Reference 600+ Potentiostat was used for all electrochemical measurements. Prior to CO electroreduction, all electrodes were pretreated at −0.7 V vs. RHE for 5 min in the argon-purged electrolyte to stabilize the surface conditions. CO gas was subsequently delivered into the electrolyte at a flow rate of 10.00 cm^3^ min^−1^ using a mass flow controller (MKS Instruments, Inc.) and calibrated using Agilent ADM flow meter. The headspace gas was vented directly into the sampling loop of a gas chromatograph (Agilent 7890B) and quantified every 17 min for gas-phase products. Liquid products were quantified by a Bruker AVIII 400 MHz NMR spectrometer. The NMR sample was prepared by mixing 500 µL of the electrolyte with 100 µL of D_2_O (Sigma-Aldrich, 99.9%) and 0.05 mM dimethyl sulfoxide (Alfa Aesar, ≥99.9%) as an internal standard. The water signal was suppressed using the excitation sculpting method. The uncompensated resistance (*R*_u_) was determined by potentiostatic electrochemical impedance spectroscopy. The potentiostat compensated for 85% of *R*_u_ during the electrolysis and the remaining 15% was manually corrected afterward to arrive at the actual potentials.

### Reactivity plot

Each reactivity data point was the average of at least three independent electrolysis experiments, based on which the SD was calculated. The electrolysis time was at least 1 h. The electrolyte pH was measured before and after each experiment, to ensure a constant electrolyte pH during the course of the electrolysis. In the partial pressure studies, the different CO partial pressures were achieved by mixing CO and Ar gases at desired ratios using mass flow controllers after calibration. One single polycrystalline Cu powder electrode was used for the CO partial pressure dependence study to eliminate the variations between the different electrodes. A 10 min electrolysis was conducted at each CO partial pressure starting from 1 atm. Then, the reaction products were sampled and the electrolysis was performed with the subsequent CO partial pressure.

### In situ SEIRAS experiments

The Cu MPs electrode for SEIRAS was prepared on Au film that was pre-deposited onto a silicon attenuated total reflection (ATR) crystal by chemical deposition as described before^16,60^. The Cu MPs electrode was prepared through dropping the Cu MPs ink solution onto the Au film. A Nafion membrane-separated two-compartment, three-electrode spectroelectrochemical flow cell was employed for the in situ SEIRAS test. The cell was integrated into the Aglient Technologies Cary 660 FTIR spectrometer equipped with a liquid nitrogen-cooled mercury cadmium telluride (MCT) detector. All spectra were collected at a 4 cm^−1^ spectral resolution and were presented in absorbance units. The potential on the cell was supplied by Solartron 1260/1287 system for electrochemical measurements. Before collecting the spectra, the background was taken at −0.2 V_SHE_ in Ar-saturated NaH_2_PO_4_ + Na_2_HPO_4_ (pH 7.2). During the test at −0.8 V_SHE_, the CO-saturated electrolytes with different pH values were delivered into the cathodic compartment using a pump, to realize the switch among different electrolytes.

### In situ SERS experiments

The Cu MPs electrode for SERS was prepared by drop-casting the above Cu MPs ink solution onto a piece of carbon fiber paper with the diameter of 1 cm. In situ SERS tests were conducted in a three-electrode spectroelectrochemical cell as shown in our recent work^53^. Before the measurements, Au@SiO_2_ were deposited onto the Cu MPs/carbon paper electrode, to enhance the Raman signal on the Cu MPs. The synthesis method for Au@SiO_2_ nanoparticles can be found in previous reports^51,61^. During the tests, the CO-saturated electrolyte was delivered into the cathodic compartment using an high-performance liquid chromatography pump to replenish CO, switch electrolyte pH, and remove the generated hydrogen bubbles to avoid the interfere with Raman signal. The electrochemical tests were conducted using a potentiostat (Princeton Applied Research, VersaSTAT 3). The Raman tests were performed on a LabRAM HR Evolution microscope (Horiba Jobin Yvon) equipped with a 633 nm He-Ne laser, a ×50 objective, a monochromator (600 grooves/mm grating), and a charge-coupled device detector. The signal acquisition time is 90 s for each Raman spectrum.

## Supplementary information

Supplementary Information

## Data Availability

All relevant data are available from the corresponding authors on reasonable request.
