# Supplementary material for: Electrokinetic and in situ spectroscopic investigations of CO electrochemical reduction on copper
Source: Nat Commun. 2021 Jun 1;12:3264. doi: 10.1038/s41467-021-23582-2 (PMC8169934; doi:10.1038/s41467-021-23582-2)
Supplement: Supplementary file 1 — Supplementary Information [file 41467_2021_23582_MOESM1_ESM.pdf]

Supplementary Information

Electrokinetic and in situ spectroscopic investigations of CO electrochemical reduction on copper

Jing Li[1], Xiaoxia Chang[2], Haochen Zhang[1], Arnav S. Malkani[2], Mu-jeng Cheng[3], Bingjun Xu[2,4] & Qi Lu[1]

[1]State Key Laboratory of Chemical Engineering, Department of Chemical Engineering, Tsinghua University, Beijing 100084, China

[2]Center for Catalytic Science and Technology, Department of Chemical and Biomolecular Engineering, University of Delaware, Newark, Delaware 19716, United States

[3]Department of Chemistry, National Cheng Kung University, Tainan 701, Taiwan College of Chemistry and Molecular Engineering, Peking University, Beijing, 100871, China

[4]College of Chemistry and Molecular Engineering, Peking University, Beijing, 100871, China

Emails: b_xu@pku.edu.cn; luqicheme@mail.tsinghua.edu.cn

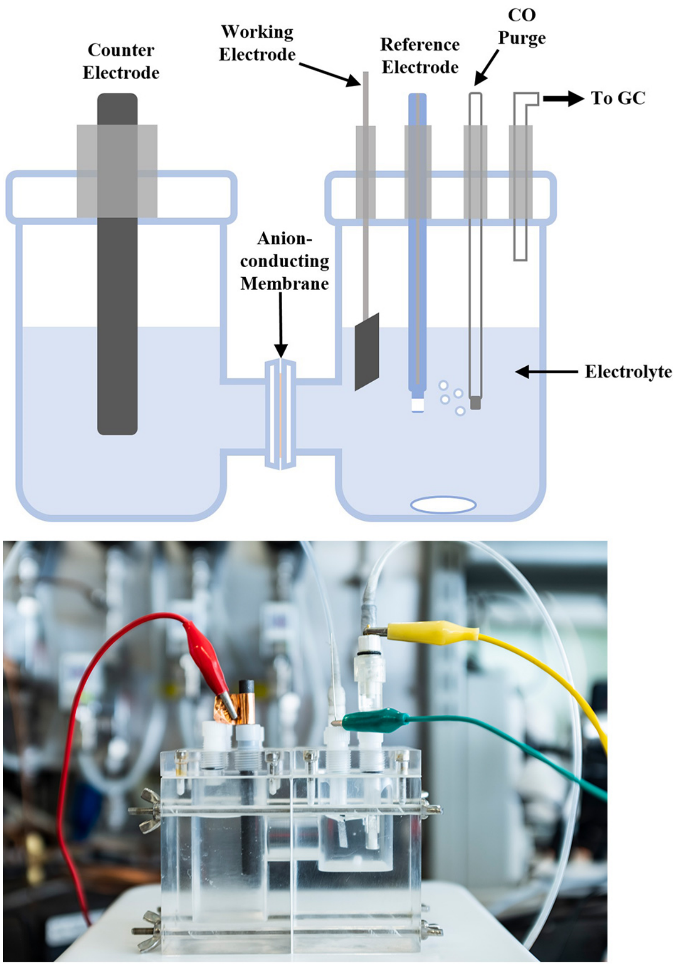

Supplementary Figure 1. Image of the three-electrode H-cell employed for CO electrolysis study.

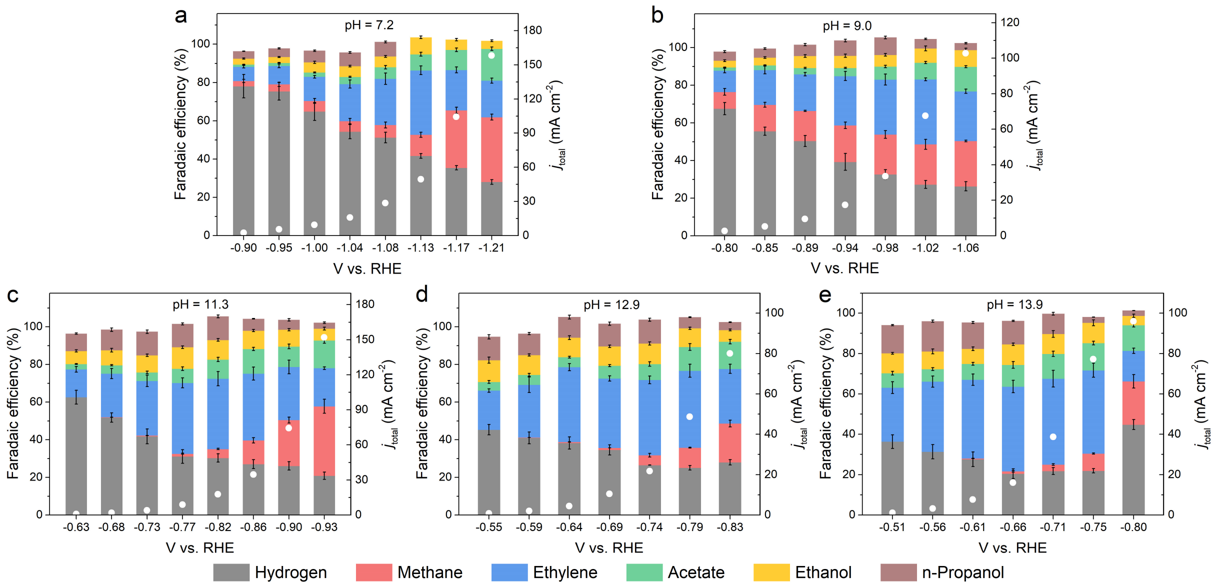

Supplementary Figure 2. Total current density and Faradaic efficiency of CO electroreduction in the electrolyte pH range of 7.2 – 13.9: (a) 0.3 M NaH_2PO_4 + 0.35 M Na_2HPO_4 (pH = 7.2), (b) 1.0 M $NaHCO_3$ (pH = 9.0), (c) 0.5 M Na_2CO_3 (pH = 11.3), (d) 0.1 M NaOH + 0.9 M $NaClO_4$ (pH = 12.9) and (e) 1.0 M NaOH (pH = 13.9). The error bars represent the standard deviation from at least three independent measurements.

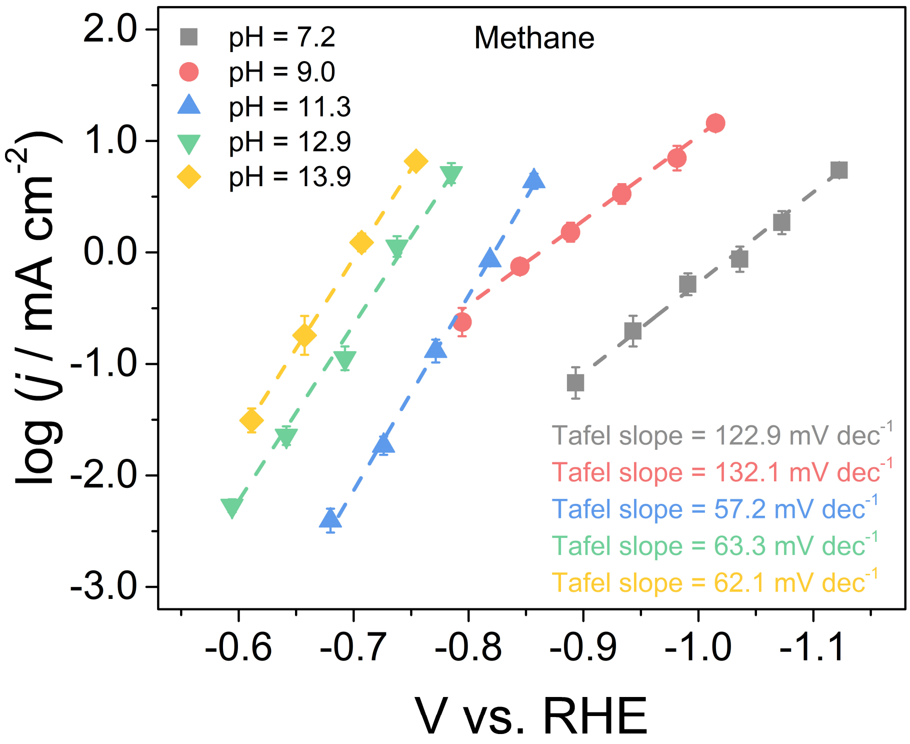

Supplementary Figure 3. The logarithms of partial current densities for CH_4 formation vs calculated RHE potential using the local pH near electrode surface (Details are shown in Supplementary Note 2). The error bars represent the standard deviation from at least three independent measurements.

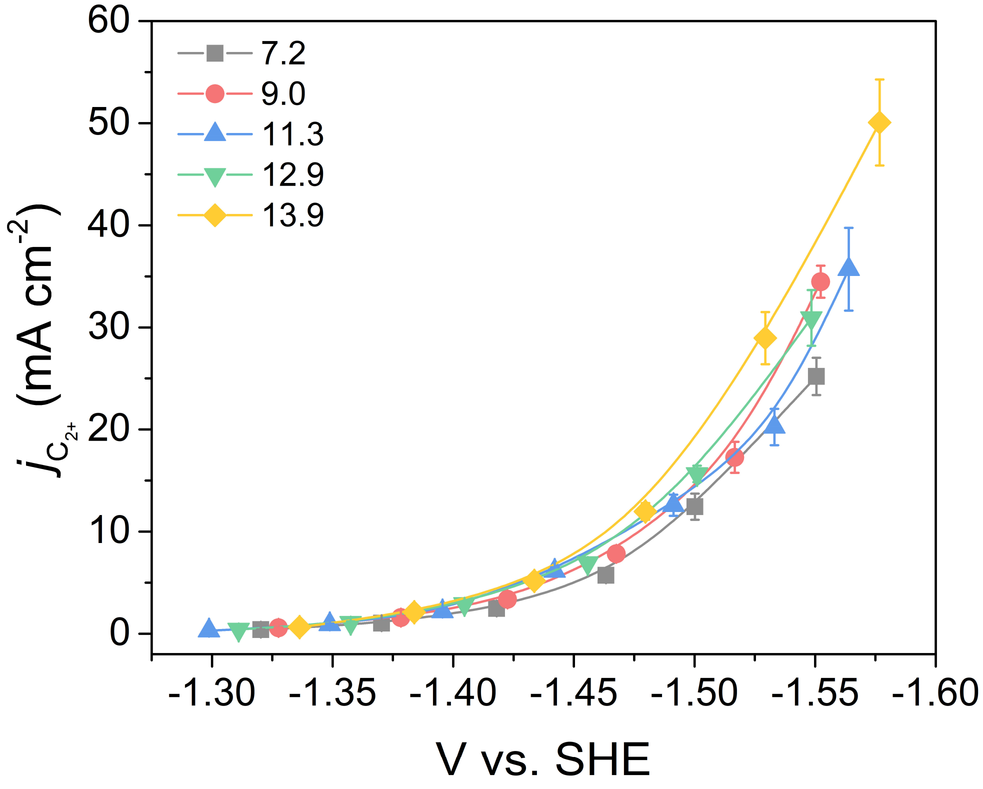

Supplementary Figure 4. Current densities for C_{2+} products formation at different electrolyte pH plotted in SHE scale. The error bars represent the standard deviation from at least three independent measurements.

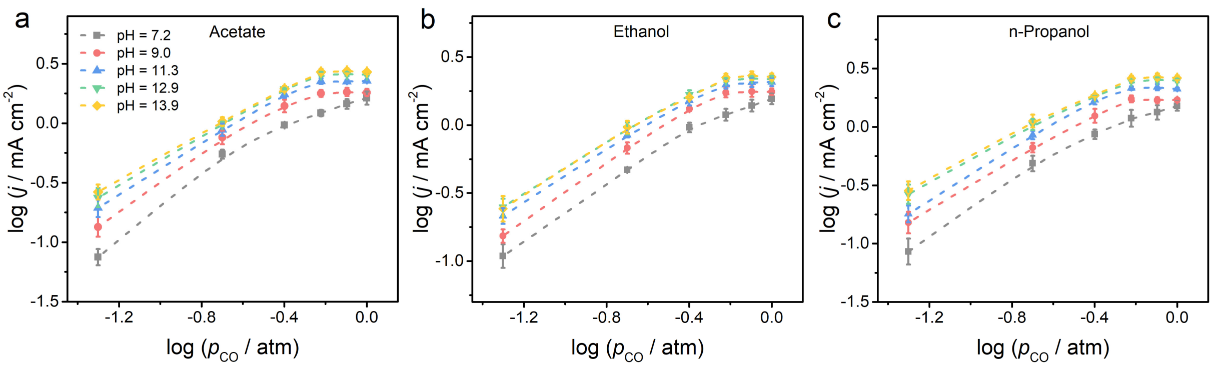

Supplementary Figure 5. p_{CO} dependent C_{2+} products formation rate at different electrolyte pH. The logarithms of partial current densities for (a) acetate, (b) ethanol and (c) n-propanol vs logarithms of p_{CO}. The potential for all electrolysis is kept at -1.50 V vs SHE. The potential for all electrolysis is kept at -1.50 V vs SHE. The error bars represent the standard deviation from at least three independent measurements.

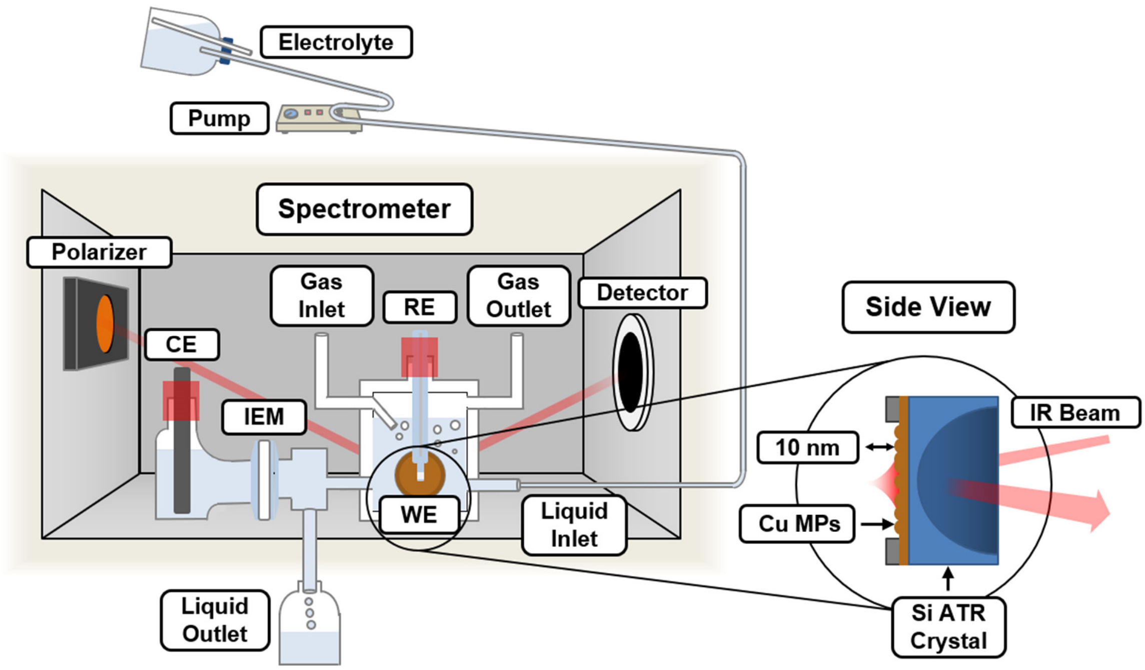

Supplementary Figure 6. Schematic of the in situ electrochemical spectroscopic cell for ATR-SEIRAS test.

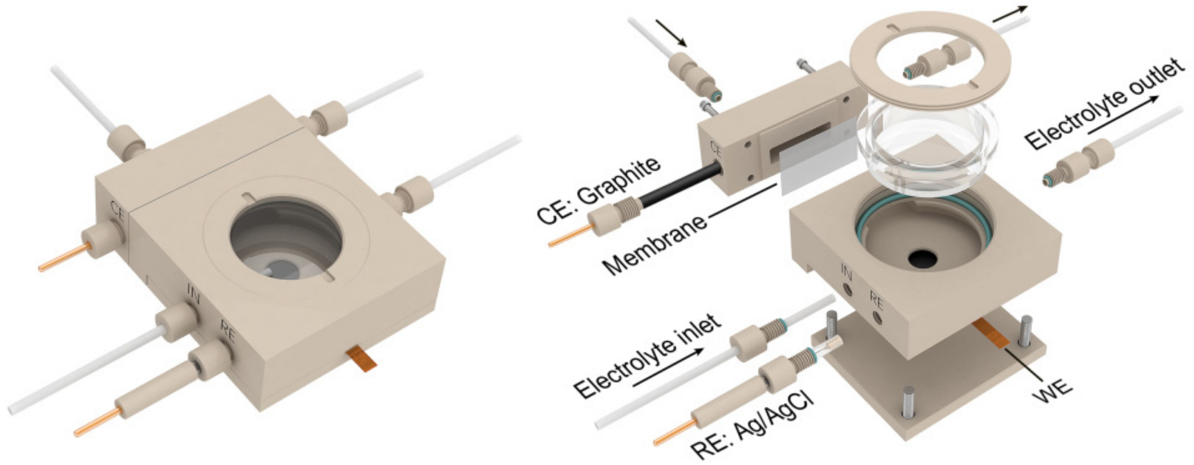

Supplementary Figure 7. Image and design of the in-situ electrochemical spectroscopic cell for SERS test.

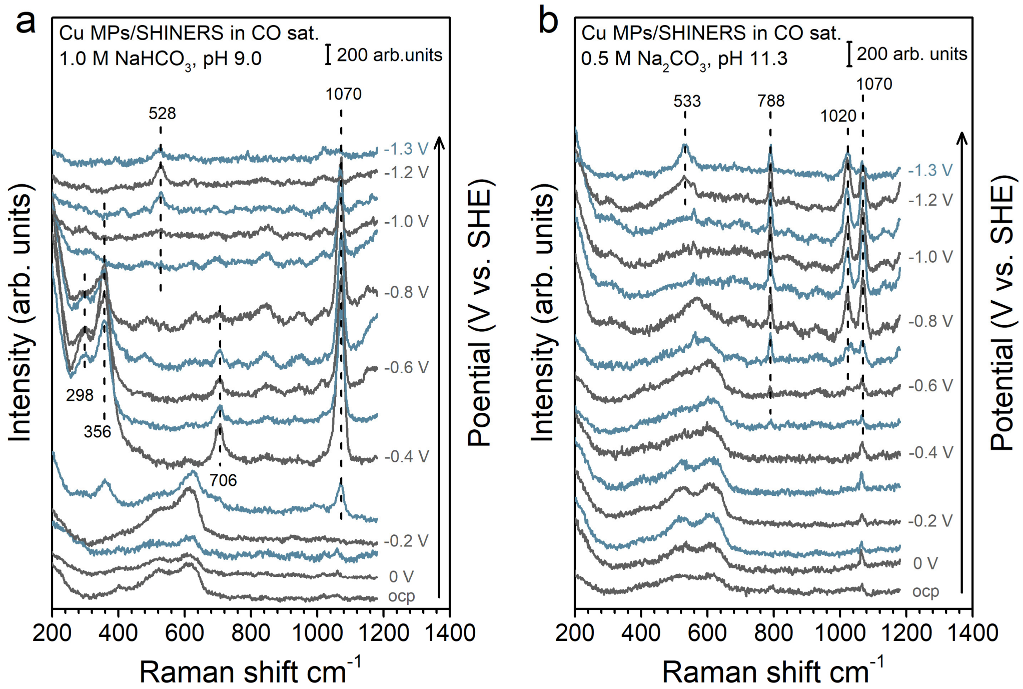

Supplementary Figure 8. SHINER spectra on Cu MPs in (a) 1.0 M $NaHCO_3$ (pH 9.0) and (b) 0.5 M Na_2CO_3 (pH 11.3) at potentials indicated in the figure. The spectra were collected at constant potentials with 0.1 V_{SHE} intervals in the cathodic direction from the OCP to -1.3 V_{SHE}.

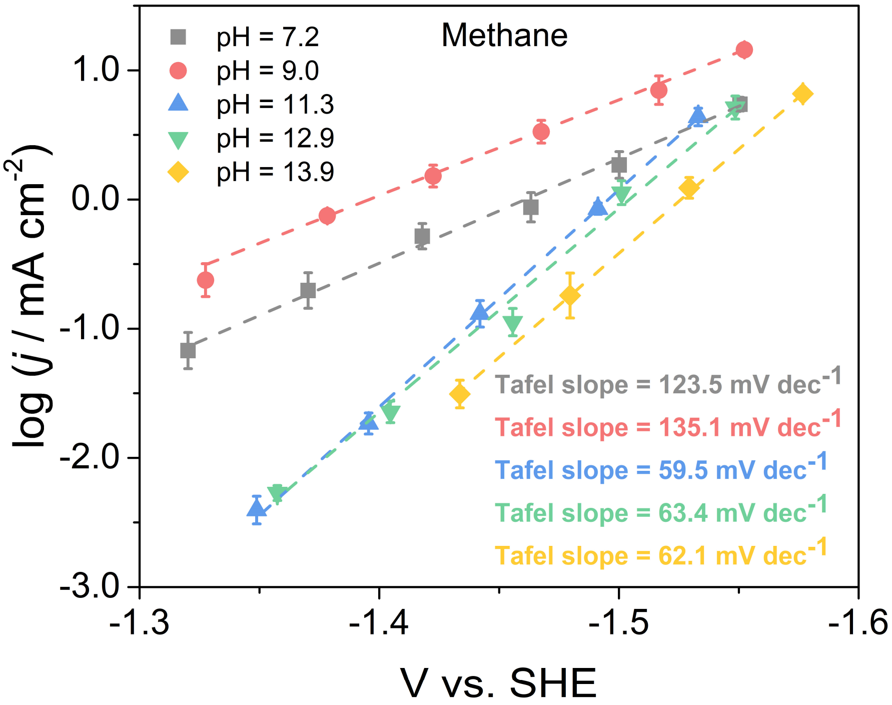

Supplementary Figure 9. The logarithms of partial current densities for CH_4 formation plotted in SHE scale at different electrolyte pH. The error bars represent the standard deviation from at least three independent measurements.

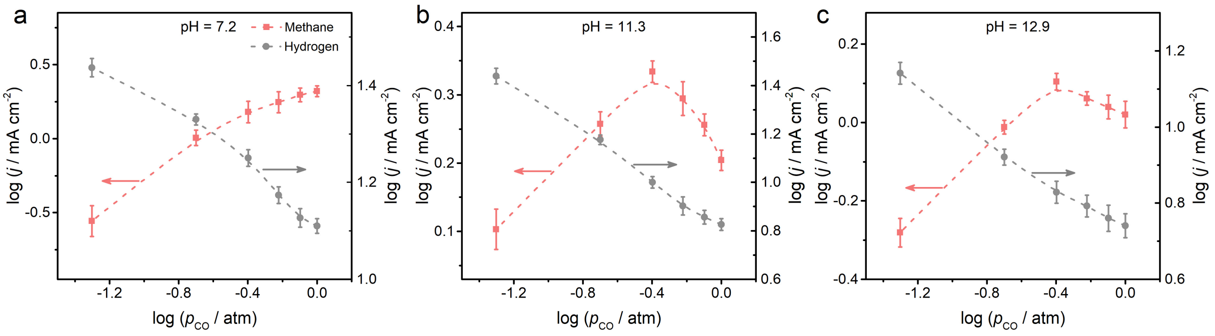

Supplementary Figure 10. p_{CO} dependent methane formation and hydrogen evolution at different electrolyte pH. The logarithms of partial current densities for methane formation (red) and hydrogen evolution (gray) vs logarithms of p_{CO} at electrolyte pH of (a) 7.2, (b) 11.3 and (c) 12.9. The potential for all electrolysis is kept at -1.50 V vs SHE. The error bars represent the standard deviation from at least three independent measurements.

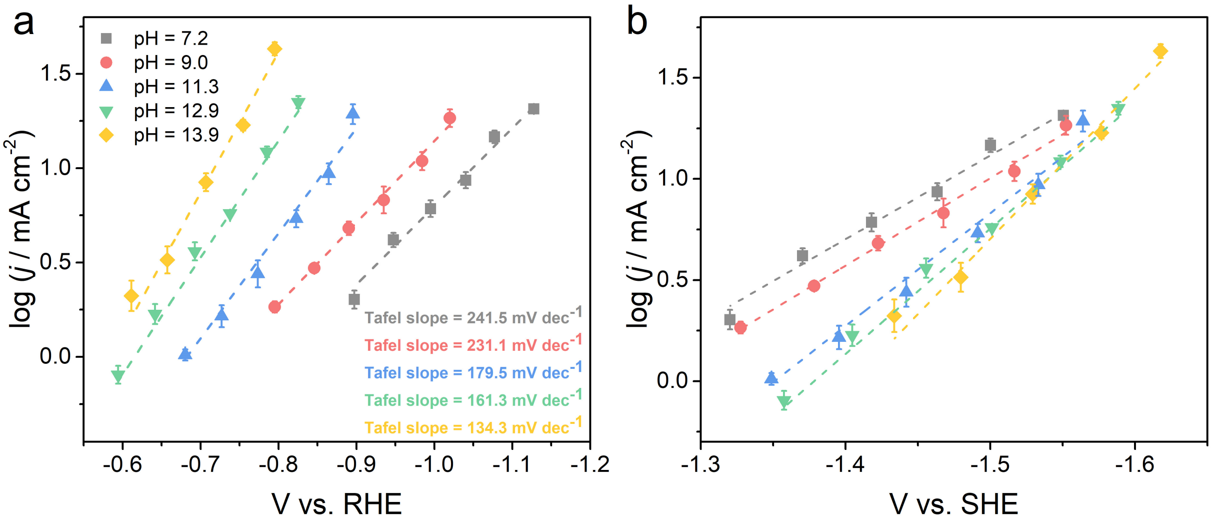

Supplementary Figure 11. The logarithms of partial current densities for hydrogen evolution plotted in (a) RHE scale and (b) SHE scale, respectively. The error bars represent the standard deviation from at least three independent measurements.

Supplementary Table 1. Summary of other possible reaction schemes for C_{2+} product (a.1-a.3) and CH_4 (b.1) formation and their corresponding Tafel slopes.

	Possible reaction scheme for C_{2+} product formation	Tafel slope	CO order at high θ_{CO}	pH dependent
a.1	$H^+ + e^- + * \rightarrow *H$<br>$*CO + *H \xrightarrow{RDS} *CO(H) + *$	59 mV dec^{-1}	negative	yes
a.2	$*CO + H_2O + e^- \rightarrow *CO(H) + OH^-$<br>$*CO + *CO(H) \xrightarrow{RDS} *C_2O_2(H) + *$<br>or $*CO(H) + *CO(H) \xrightarrow{RDS} *C_2O_2(H)_2 + *$	59 mV dec^{-1}	0	no
a.3	$*CO + H^+ + e^- \rightarrow *CO(H)$<br>$*CO + *CO(H) \xrightarrow{RDS} *C_2O_2(H) + *$<br>or $*CO(H) + *CO(H) \xrightarrow{RDS} *C_2O_2(H)_2 + *$	59 mV dec^{-1}	0	yes
	CH_4 formation			
b.1	$H^+ + e^- + * \rightarrow *H$<br>$*CO + H^+ + e^- \rightarrow *CO(H)$<br>$*CO(H) + *H \xrightarrow{RDS} *CO(H)_2 + *$	29.5 mV dec^{-1}	negative	yes

Supplementary Table 2. The estimated CO adsorption equilibrium constant based on the proposed mechanisms in this work. The rate expressions for the formation of C_{2+} products and methane are shown in Supplementary Note 1.

pH value	K_{CO}^{C2+}	$K_{CO}^{methane}$
7.2	3.9	2.6
9.0	7.6	7.0
11.3	7.6	3.2
12.9	7.3	2.2
13.9	6.8	2.2

Supplementary Note 1: Derivation of mechanistic models

Reaction schemes of C_{2+} production

Scheme 1: Dimerization between two *CO

The rate-determining step (RDS) is the coupling between two *CO.

RDS: $*CO + *CO + e^- \xrightarrow{k_{C_2O_2^-}} *C_2O_2^- + *$

The rate of this process can be written as:

$$j_{C_{2+}} = k_{C_2O_2^-}[*CO]^2 \exp\left(\frac{\beta\eta F}{RT}\right) \quad (1)$$

We assume a fast equilibrium for CO adsorption on the surface, where the equilibrium constant is K_{CO}:

$$CO(g) + * \xleftrightarrow{K_{CO}} *CO$$

$$K_{CO} = \frac{[*CO]}{P_{CO}[*]} \quad (2)$$

Because *CO is the only major adsorbate, the concentration of all sites $[L]$ can be expressed as:

$$[L] = [*CO] + [*] \quad (3)$$

From equations (2) and (3), we can obtain:

$$[*CO] = \frac{K_{CO}P_{CO}}{1+K_{CO}P_{CO}}[L] \quad (4)$$

Combining equations (1) and (4) yields:

$$j_{C_{2+}} = k_{C_2O_2^-} \exp\left(\frac{\beta\eta F}{RT}\right)\left(\frac{K_{CO}P_{CO}}{1+K_{CO}P_{CO}}\right)^2 [L]^2 \quad (5)$$

At low P_{CO}, according to equation (4), the $[*CO]$ term approaches $K_{CO}P_{CO}[L]$, and the rate expression can be expressed as:

$$j_{C_{2+}} = k_{C_2O_2^-} \exp\left(\frac{\beta\eta F}{RT}\right) K_{CO}{}^2 P_{CO}{}^2 [L]^2 \quad (6)$$

The reaction order with respect to P_{CO} is 2.

At high P_{CO}, the $[*CO]$ term approaches $[L]$, and the rate expression becomes:

$$j_{C_{2+}} = k_{C_2O_2^-} \exp\left(\frac{\beta\eta F}{RT}\right)[L]^2 \tag{7}$$

The reaction order with respect to P_{CO} is 0.

This reaction scheme can be consistent with the experimental observations shown in Figs. 2a and Supplementary Fig. 5.

Scheme 2: Dimerization between *CO and CO$_{(b)}$

The RDS is the coupling between *CO and a CO molecule:

RDS: $*CO + CO_{(b)} + e^- \xrightarrow{k_{C_2O_2^-}} *C_2O_2^-$

The rate expression can be written as:

$$j_{C_{2+}} = k_{C_2O_2^-}[*CO]P_{CO} \exp\left(\frac{\beta\eta F}{RT}\right) \tag{8}$$

We assume a fast equilibrium for CO adsorption on the surface, where the equilibrium constant is K_{CO}:

$$CO(g) + * \xleftrightarrow{K_{CO}} *CO$$

$$K_{CO} = \frac{[*CO]}{P_{CO}[*]} \tag{9}$$

Because *CO is the only major adsorbate, the concentration of all sites $[L]$ can be expressed as:

$$[L] = [*CO] + [*] \tag{10}$$

From equations (9) and (10), we can obtain:

$$[*CO] = \frac{K_{CO}P_{CO}}{1+K_{CO}P_{CO}}[L] \tag{11}$$

Combining equations (8) and (11) yields:

$$j_{C_{2+}} = k_{C_2O_2^-} \exp\left(\frac{\beta\eta F}{RT}\right)\frac{K_{CO}P_{CO}^{\ 2}}{1+K_{CO}P_{CO}}[L] \tag{12}$$

At low P_{CO}, according to equation (11), the $[*CO]$ term approaches $K_{CO}P_{CO}[L]$, and the rate expression can be expressed as:

$$j_{C_{2+}} = k_{C_2O_2^-} \exp\left(\frac{\beta\eta F}{RT}\right)K_{CO}P_{CO}^{\ 2}[L] \tag{13}$$

The reaction order with respect to P_{CO} is 2.

At high P_{CO}, the $[*CO]$ term approaches $[L]$, and the rate expression becomes:

$$j_{C_{2+}} = k_{C_2O_2^-} \exp\left(\frac{\beta\eta F}{RT}\right) P_{CO}[L] \tag{14}$$

The reaction order with respect to P_{CO} is 1.

This reaction scheme is not consistent with the experimental observations shown in Fig. 2a and Supplementary Fig. 5.

Scheme 3: Dimerization between *CO(H) and *CO

Possibility 1: The RDS is the hydrogenation of surface-bound CO (noted as *CO). The hydrogen source is the proton in solution. C_{2+} is produced via CO coupling between *CO and *CO(H).

RDS: $*CO + H^+ + e^- \xrightarrow{k_{CO(H)}} *CO(H)$

C_{2+} production: $*CO + *CO(H) \xrightarrow{k_{C_2O_2(H)}} *C_2O_2\text{(H)}+*$

Note that *CO(H) is also an important intermediate for methane formation.

Methane production: $*CO(H) \xrightarrow{k_{CH_4}} \longrightarrow CH_4$

Assuming $[*CO(H)]$ remains constant under steady state, we can obtain:

$$j_{C_{2+}} = k_{CO(H)}[*CO][H^+]\exp\left(\frac{\beta\eta F}{RT}\right) \tag{15}$$

From equation (15), we can obtain:

$$[*CO(H)] = \frac{k_{CO(H)}[*CO][H^+]\exp\left(\frac{\beta\eta F}{RT}\right)}{k_{C_2O_2(H)}[*CO] + k_{CH_4}} \tag{16}$$

The rate expression of C_{2+} formation can be written as:

$$j_{C_{2+}} = k_{C_2O_2(H)}[*CO][*CO(H)] = \frac{k_{C_2O_2(H)}k_{CO(H)}[*CO]^2[H^+]\exp\left(\frac{\beta\eta F}{RT}\right)}{k_{C_2O_2(H)}[*CO] + k_{CH_4}} \tag{17}$$

We assume a fast equilibrium for CO adsorption on the surface, where the equilibrium constant is K_{CO}:

$$CO(g) + * \xleftrightarrow{K_{CO}} *CO$$

$$K_{CO} = \frac{[*CO]}{P_{CO}[*]} \tag{18}$$

$[*CO(H)]$ is small due to its consumption being faster than its production. Therefore, *CO is the only major adsorbate and the concentration of all sites $[L]$ can be expressed as:

$$[L] = [*CO] + [*] \tag{19}$$

From equations (18) and (19), we can obtain:

$$[*CO] = \frac{K_{CO}P_{CO}}{1+K_{CO}P_{CO}}[L] \tag{20}$$

Combining equations (17) and (20) yields:

$$j_{C_{2+}} = \frac{k_{C_2O_2(H)}k_{CO(H)}K_{CO}{}^2P_{CO}{}^2[H^+]\exp\left(\frac{\beta\eta F}{RT}\right)[L]^2}{k_{C_2O_2(H)}K_{CO}P_{CO}\left(1+K_{CO}P_{CO}\right)[L] + k_{CH_4}\left(1+K_{CO}P_{CO}\right)^2} \tag{21}$$

At low P_{CO}, according to equation (20), the $[*CO]$ term approaches $K_{CO}P_{CO}[L]$, and the rate expression can be expressed as:

$$j_{C_{2+}} = \frac{k_{C_2O_2(H)}k_{CO(H)}}{k_{CH_4}}K_{CO}{}^2P_{CO}{}^2[H^+]\exp\left(\frac{\beta\eta F}{RT}\right)[L]^2 \tag{22}$$

The reaction order with respect to P_{CO} is 2.

At high P_{CO}, the $[*CO]$ term approaches $[L]$, and the rate expression becomes:

$$j_{C_{2+}} = \frac{k_{C_2O_2(H)}k_{CO(H)}[H^+]\exp\left(\frac{\beta\eta F}{RT}\right)[L]^2}{k_{C_2O_2(H)}[L] + k_{CH_4}} \tag{23}$$

The reaction order with respect to P_{CO} is 0.

This reaction scheme can be consistent with the experimental observations shown in Fig. 2a and Supplementary Fig. 5. However, it leads to a pH dependence of C_{2+} products formation which is inconsistent with our observation shown in Fig. 1 that rates of C_{2+} products are pH independent.

Possibility 2: The RDS is the hydrogenation of surface-bound CO (noted as *CO). The hydrogen source is the water in solution. C_{2+} is produced via CO coupling between *CO and *CO(H).

RDS: $*CO + H_2O + e^- \xrightarrow{k_{CO(H)}} *CO(H) + OH^-$

C_{2+} production: $*CO + *CO(H) \xrightarrow{k_{C_2O_2(H)}} *C_2O_2\text{(H)}+*$

Note that *CO(H) is also an important intermediate for methane formation.

Methane production: $*CO(H) \xrightarrow{k_{CH_4}} \longrightarrow CH_4$

Assuming $[*CO(H)]$ remains constant under steady state, we can obtain:

$$k_{CO(H)}[*CO]\exp\left(\frac{\beta\eta F}{RT}\right) = k_{C_2O_2(H)}[*CO][*CO(H)] + k_{CH_4}[*CO(H)] \tag{24}$$

From equation (24), we can obtain:

$$[*CO(H)] = \frac{k_{CO(H)}[*CO]\exp\left(\frac{\beta\eta F}{RT}\right)}{k_{C_2O_2(H)}[*CO] + k_{CH_4}} \tag{25}$$

The rate expression of C_{2+} formation can be written as:

$$j_{C_{2+}} = k_{C_2O_2(H)}[*CO][*CO(H)] = \frac{k_{C_2O_2(H)}k_{CO(H)}[*CO]^2 \exp\left(\frac{\beta\eta F}{RT}\right)}{k_{C_2O_2(H)}[*CO] + k_{CH_4}} \tag{26}$$

We assume a fast equilibrium for CO adsorption on the surface, where the equilibrium constant is K_{CO}:

$$CO(g) + * \xleftrightarrow{K_{CO}} *CO$$

$$K_{CO} = \frac{[*CO]}{P_{CO}[*]} \tag{27}$$

$[*CO(H)]$ is small due to its consumption being faster than its production. Therefore, *CO is the only major adsorbate and the concentration of all sites $[L]$ can be expressed as:

$$[L] = [*CO] + [*] \tag{28}$$

From equations (27) and (28), we can obtain:

$$[*CO] = \frac{K_{CO}P_{CO}}{1+K_{CO}P_{CO}}[L] \tag{29}$$

Combining equations (26) and (29) yields:

$$j_{C_{2+}} = \frac{k_{C_2O_2(H)}k_{CO(H)}K_{CO}{}^2P_{CO}{}^2 \exp\left(\frac{\beta\eta F}{RT}\right)[L]^2}{k_{C_2O_2(H)}K_{CO}P_{CO}\left(1 + K_{CO}P_{CO}\right)[L] + k_{CH_4}\left(1 + K_{CO}P_{CO}\right)^2} \tag{30}$$

At low P_{CO}, according to equation (29), the $[*CO]$ term approaches $K_{CO}P_{CO}[L]$, and the rate expression can be expressed as:

$$j_{C_{2+}} = \frac{k_{C_2O_2(H)}k_{CO(H)}}{k_{CH_4}} K_{CO}{}^2 P_{CO}{}^2 \exp\left(\frac{\beta\eta F}{RT}\right)[L]^2 \tag{31}$$

The reaction order with respect to P_{CO} is 2.

At high P_{CO}, the $[*CO]$ term approaches $[L]$, and the rate expression becomes:

$$j_{C_{2+}} = \frac{k_{C_2O_2(H)}k_{CO(H)} \exp\left(\frac{\beta\eta F}{RT}\right)[L]^2}{k_{C_2O_2(H)}[L] + k_{CH_4}} \tag{32}$$

The reaction order with respect to P_{CO} is 0.

This reaction scheme can be consistent with the experimental observations shown in Fig. 2a and Supplementary Fig. 5. It leads to a pH independence of C_{2+} products formation which is consistent with our observation shown in Fig. 1 that rates of C_{2+} products are pH independent.

Scheme 4: Dimerization between two *CO(H)

Possibility 1: The RDS is the hydrogenation of surface-bound CO (noted as *CO). **The hydrogen source is the proton in solution.** C_{2+} is produced via CO coupling between two *CO(H).

RDS: $*CO + H^+ + e^- \xrightarrow{k_{CO(H)}} *CO(H)$

C_{2+} production: $*CO(H) + *CO(H) \xrightarrow{k_{C_2O_2(H)_2}} *C_2O_2(\mathrm{H})_2 + *$

Note that *CO(H) is also an important intermediate for methane formation.

Methane production: $*CO(H) \xrightarrow{k_{CH_4}} \longrightarrow CH_4$

Assuming $[*CO(H)]$ remains constant under steady state, we can obtain:

$$k_{CO(H)}[*CO][H^+]\exp\left(\frac{\beta\eta F}{RT}\right) = k_{C_2O_2(H)_2}[*CO(H)]^2 + k_{CH_4}[*CO(H)] \tag{33}$$

From equation (33), we can obtain:

$$[*CO(H)] = \frac{\sqrt{k_{CH_4}{}^2 + 4k_{C_2O_2(H)_2}k_{CO(H)}[*CO][H^+]\exp\left(\frac{\beta\eta F}{RT}\right)} - k_{CH_4}}{2k_{C_2O_2(H)_2}} \tag{34}$$

The rate expression of C_{2+} formation can be written as:

$$j_{C_{2+}} = k_{C_2O_2(H)_2}[*CO(H)]^2 = k_{C_2O_2(H)_2}\left(\frac{\sqrt{k_{CH_4}{}^2 + 4k_{C_2O_2(H)_2}k_{CO(H)}[*CO][H^+]\exp\left(\frac{\beta\eta F}{RT}\right)} - k_{CH_4}}{2k_{C_2O_2(H)_2}}\right)^2 \quad (35)$$

We assume a fast equilibrium for CO adsorption on the surface, where the equilibrium constant is K_{CO}:

$$CO(g) + * \xleftrightarrow{K_{CO}} *CO$$

$$K_{CO} = \frac{[*CO]}{P_{CO}[*]} \quad (36)$$

$[*CO(H)]$ is small due to its consumption being faster than its production. Therefore, *CO is the only major adsorbate and the concentration of all sites $[L]$ can be expressed as:

$$[L] = [*CO] + [*] \quad (37)$$

From equations (36) and (37), we can obtain:

$$[*CO] = \frac{K_{CO}P_{CO}}{1+K_{CO}P_{CO}}[L] \quad (38)$$

Combining equations (35) and (38) yields:

$$j_{C_{2+}} = k_{C_2O_2(H)_2}\left(\frac{\sqrt{k_{CH_4}{}^2 + 4k_{C_2O_2(H)_2}k_{CO(H)}\frac{K_{CO}P_{CO}}{1+K_{CO}P_{CO}}[L][H^+]\exp\left(\frac{\beta\eta F}{RT}\right)} - k_{CH_4}}{2k_{C_2O_2(H)_2}}\right)^2 \quad (39)$$

At low P_{CO}, according to equation (38), the $[*CO]$ term approaches $K_{CO}P_{CO}[L]$, and the rate expression can be expressed as:

$$j_{C_{2+}} = k_{C_2O_2(H)_2}\left(\frac{\sqrt{k_{CH_4}{}^2 + 4k_{C_2O_2(H)_2}k_{CO(H)}K_{CO}P_{CO}[L][H^+]\exp\left(\frac{\beta\eta F}{RT}\right)} - k_{CH_4}}{2k_{C_2O_2(H)_2}}\right)^2 \quad (40)$$

The reaction order with respect to P_{CO} is close to 1.

At high P_{CO}, the $[*CO]$ term approaches $[L]$, and the rate expression becomes:

$$j_{C_{2+}} = k_{C_2O_2(H)_2}\left(\frac{\sqrt{k_{CH_4}{}^2 + 4k_{C_2O_2(H)_2}k_{CO(H)}[L][H^+]\exp\left(\frac{\beta\eta F}{RT}\right)} - k_{CH_4}}{2k_{C_2O_2(H)_2}}\right)^2 \quad (41)$$

The reaction order with respect to P_{CO} is 0.

This reaction scheme can be consistent with the experimental observations shown in Fig. 2a and Supplementary Fig. 5. However, it leads to a pH dependence of C_{2+} products formation which is inconsistent with our observation shown in Fig. 1 that rates of C_{2+} products are pH independent.

Possibility 2: The RDS is the hydrogenation of surface-bound CO (noted as *CO). **The hydrogen source is the water in solution.** C_{2+} is produced via CO coupling between two *CO(H).

RDS: $*CO + H_2O + e^- \xrightarrow{k_{CO(H)}} *CO(H) + OH^-$

C_{2+} production: $*CO(H) + *CO(H) \xrightarrow{k_{C_2O_2(H)_2}} *C_2O_2(\mathrm{H})_2 + *$

Note that *CO(H) is also an important intermediate for methane formation.

Methane production: $*CO(H) \xrightarrow{k_{CH_4}} \longrightarrow CH_4$

Assuming $[*CO(H)]$ remains constant under steady state, we can obtain:

$$k_{CO(H)}[*CO]\exp\left(\frac{\beta\eta F}{RT}\right) = k_{C_2O_2(H)_2}[*CO(H)]^2 + k_{CH_4}[*CO(H)] \quad (42)$$

From equation (42), we can obtain:

$$[*CO(H)] = \frac{\sqrt{k_{CH_4}{}^2 + 4k_{C_2O_2(H)_2}k_{CO(H)}[*CO]\exp\left(\frac{\beta\eta F}{RT}\right)} - k_{CH_4}}{2k_{C_2O_2(H)_2}} \quad (43)$$

The rate expression of C_{2+} formation can be written as:

$$j_{C_{2+}} = k_{C_2O_2(H)_2}[*CO(H)]^2 = k_{C_2O_2(H)_2}\left(\frac{\sqrt{k_{CH_4}{}^2 + 4k_{C_2O_2(H)_2}k_{CO(H)}[*CO]\exp\left(\frac{\beta\eta F}{RT}\right)} - k_{CH_4}}{2k_{C_2O_2(H)_2}}\right)^2 \quad (44)$$

We assume a fast equilibrium for CO adsorption on the surface, where the equilibrium constant is K_{CO}:

$$CO(g) + * \xleftrightarrow{K_{CO}} *CO$$

$$K_{CO} = \frac{[*CO]}{P_{CO}[*]} \tag{45}$$

$[*CO(H)]$ is small due to its consumption being faster than its production. Therefore, *CO is the only major adsorbate and the concentration of all sites $[L]$ can be expressed as:

$$[L] = [*CO] + [*] \tag{46}$$

From equations (45) and (46), we can obtain:

$$[*CO] = \frac{K_{CO}P_{CO}}{1+K_{CO}P_{CO}}[L] \tag{47}$$

Combining equations (44) and (47) yields:

$$j_{C_{2+}} = k_{C_2O_2(H)_2}\left(\frac{\sqrt{k_{CH_4}{}^2 + 4k_{C_2O_2(H)_2}k_{CO(H)}\frac{K_{CO}P_{CO}}{1+K_{CO}P_{CO}}[L]\exp\left(\frac{\beta\eta F}{RT}\right)} - k_{CH_4}}{2k_{C_2O_2(H)_2}}\right)^2 \tag{48}$$

At low P_{CO}, according to equation (47), the $[*CO]$ term approaches $K_{CO}P_{CO}[L]$, and the rate expression can be expressed as:

$$j_{C_{2+}} = k_{C_2O_2(H)_2}\left(\frac{\sqrt{k_{CH_4}{}^2 + 4k_{C_2O_2(H)_2}k_{CO(H)}K_{CO}P_{CO}[L]\exp\left(\frac{\beta\eta F}{RT}\right)} - k_{CH_4}}{2k_{C_2O_2(H)_2}}\right)^2 \tag{49}$$

The reaction order with respect to P_{CO} is close to 1.

At high P_{CO}, the $[*CO]$ term approaches $[L]$, and the rate expression becomes:

$$j_{C_{2+}} = k_{C_2O_2(H)_2}\left(\frac{\sqrt{k_{CH_4}{}^2 + 4k_{C_2O_2(H)_2}k_{CO(H)}[L]\exp\left(\frac{\beta\eta F}{RT}\right)} - k_{CH_4}}{2k_{C_2O_2(H)_2}}\right)^2 \tag{50}$$

The reaction order with respect to P_{CO} is 0.

This reaction scheme can be consistent with the experimental observations shown in Fig. 2a and Supplementary Fig. 5. It also leads to a pH independence of C_{2+} products formation which is consistent with our observation shown in Fig. 1 that rates of C_{2+} products are pH independent.

Reaction schemes of methane production

Scheme 1: Coupling between *CO and *H.

Possibility 1: The RDS is the protonation of *CO. The proton source is the surface adsorbed *H (*B.1* mechanism).

RDS: $*CO + *H \xrightarrow{k_{CO(H)}} *CO(H) + *$

The rate expression can be written as:

$$j_{CH_4} = k_{CO(H)}[*CO][*H] \quad (51)$$

We assume a fast equilibrium for CO adsorption on the surface, where the equilibrium constant is K_{CO}:

$$CO(g) + * \xleftrightarrow{K_{CO}} *CO$$

$$K_{CO} = \frac{[*CO]}{P_{CO}[*]} \quad (52)$$

Similarly, the reduction of H_2O to produce *H is assumed to be a fast equilibrium. We note that the absolute H_{ads} coverage on Cu likely remains low due to the lack of experimental observation and relatively low hydrogen binding energy.[1] The H_{ads} coverage keep nearly constant with the change of applied potential:

$$H_2O + e^- + * \xleftrightarrow{K_H} *H + OH^-$$

$$K_H = \frac{K_W[*H]}{[H^+][*]} \quad (53)$$

Where K_w represents the ion-product constant of water.

The concentration of all sites $[L]$ can be expressed as:

$$[L] = [*CO] + [*H] + [*] \quad (54)$$

The coverage of H_{ads} is typically considered to be small:

$$[L] = [*CO] + [*] \quad (55)$$

From equations (52), (53) and (55), we can obtain:

$$[*CO] = \frac{K_{CO}P_{CO}}{1+K_{CO}P_{CO}}[L] \quad (56)$$

$$[*H] = \frac{K_H[H^+]}{K_W\left(1+K_{CO}P_{CO}\right)}[L] \quad (57)$$

Combining equations (51), (56) and (57) yields:

$$j_{CH_4} = k_{CO(H)} \frac{K_{CO} P_{CO} K_H [H^+]}{K_W (1 + K_{CO} P_{CO})^2} [L]^2 \tag{58}$$

At low P_{CO}, according to equations (56) and (57), the $[*CO]$ term approaches $K_{CO} P_{CO} [L]$, and the $[*H]$ term approaches $\frac{K_H [H^+]}{K_W} [L]$, the rate expression can be expressed as:

$$j_{CH_4} = \frac{k_{CO(H)} K_H K_{CO} P_{CO} [H^+]}{K_W} [L]^2 \tag{59}$$

The reaction order with respect to P_{CO} is 1

At high P_{CO}, the $[*CO]$ term approaches $[L]$, and the $[*H]$ term approaches $\frac{K_H [H^+]}{K_W} \frac{1}{K_{CO} P_{CO}} [L]$, the rate expression can be expressed as:

$$j_{CH_4} = \frac{k_{CO(H)} K_H [H^+]}{K_W} \frac{1}{K_{CO} P_{CO}} [L]^2 \tag{60}$$

The reaction order with respect to P_{CO} is -1

Possibility 2: The RDS is the Volmer step followed by the protonation of *CO. The proton source is the surface adsorbed *H (*B.2* mechanism).

RDS: $H_2O + e^- + * \xrightarrow[RDS]{k_H} *H + OH^-$

$$*CO + *H \xrightarrow{k_{CO(H)}} *CO(H) + *$$

The rate expression can be written as:

$$j_{CH_4} = k_{CO(H)} [*CO][*H] \tag{61}$$

We assume a fast equilibrium for CO adsorption on the surface, where the equilibrium constant is K_{CO}:

$$CO(g) + * \xleftrightarrow{K_{CO}} *CO$$

$$K_{CO} = \frac{[*CO]}{P_{CO}[*]} \tag{62}$$

the concentration of all sites $[L]$ can be expressed as:

$$[L] = [*CO] + [*H] + [*] \tag{63}$$

The coverage of H_{ads} is typically considered to be small:

$$[L]=[*CO]+[*] \tag{64}$$

From equations (62) and (64), we can obtain:

$$[*CO]=\frac{K_{CO}P_{CO}}{1+K_{CO}P_{CO}}[L] \tag{65}$$

Note that $*H$ is also an important intermediate for hydrogen evolution reaction. Hydrogen evolution reaction through the Volmer-Heyrovsky mechanism:

$$*H+H_2O+e^- \xrightarrow{k_{H2}} H_2+OH^-$$

Assuming $*H$ remains constant under steady state, we can obtain:

$$k_H[*]\exp\left(\frac{\beta\eta F}{RT}\right)=k_{CO(H)}[*CO][*H]+k_{H_2}[*H]\exp\left(\frac{\eta F}{RT}\right) \tag{66}$$

From equations (64) and (66), we can obtain:

$$[*H]=\frac{k_H\exp\left(\frac{\beta\eta F}{RT}\right)([L]-[*CO])}{k_H+k_{CO(H)}[*CO]+k_{H_2}[*H]\exp\left(\frac{\eta F}{RT}\right)} \tag{67}$$

Combining equations (65) and (67) yields:

$$[*H]=\frac{k_H\exp\left(\frac{\beta\eta F}{RT}\right)[L]}{(k_H\exp\left(\frac{\beta\eta F}{RT}\right)+k_{H_2}\exp\left(\frac{\eta F}{RT}\right))+(k_{CO(H)}[L]+k_H\exp\left(\frac{\beta\eta F}{RT}\right)+k_{H_2}\exp\left(\frac{\eta F}{RT}\right))K_{CO}P_{CO}} \tag{68}$$

Combining equations (61) and (68) yields:

$$j_{CH_4}=\frac{k_{CO(H)}k_H\exp\left(\frac{\beta\eta F}{RT}\right)K_{CO}P_{CO}[L]^2}{k_H\exp\left(\frac{\beta\eta F}{RT}\right)+k_{H_2}\exp\left(\frac{\eta F}{RT}\right)+(k_{CO(H)}[L]+2(k_H\exp\left(\frac{\beta\eta F}{RT}\right)+k_{H_2}\exp\left(\frac{\eta F}{RT}\right)))K_{CO}P_{CO}+(k_{CO(H)}[L]+k_H\exp\left(\frac{\beta\eta F}{RT}\right)+k_{H_2}\exp\left(\frac{\eta F}{RT}\right))K_{CO}{}^2P_{CO}{}^2} \tag{69}$$

At low P_{CO}, the rate expression can be expressed as:

$$j_{CH_4}=\frac{k_{CO(H)}k_HK_{CO}P_{CO}\exp\left(\frac{\beta\eta F}{RT}\right)[L]^2}{k_H\exp\left(\frac{\beta\eta F}{RT}\right)+k_{H_2}\exp\left(\frac{\eta F}{RT}\right)} \tag{70}$$

The reaction order with respect to P_{CO} is 1.

At high P_{CO}, the rate expression can be expressed as:

$$j_{CH_4} = \frac{k_{CO(H)} k_H \exp\left(\frac{\beta\eta F}{RT}\right)[L]^2}{(k_{CO(H)}[L] + k_H \exp\left(\frac{\beta\eta F}{RT}\right) + k_{H_2} \exp\left(\frac{\eta F}{RT}\right)) K_{CO} P_{CO}} \tag{71}$$

The reaction order with respect to P_{CO} is -1.

Scheme 2: Reaction between *CO and H₂O in solution.

Possibility1: The RDS is the protonation of *CO. The hydrogen source is the water in the electrolyte (*B.3* and *B.5* mechanisms).

RDS: $*CO + H_2O + e^- \xrightarrow{k_{CO(H)}} *CO(H) + OH^-$

The rate expression can be written as:

$$j_{CH_4} = k_{CO(H)}[*CO]\exp\left(\frac{\beta\eta F}{RT}\right) \tag{72}$$

where β is the symmetry factor for the reduction process.

We assume a fast equilibrium for CO adsorption on the surface, where the equilibrium constant is K_{CO}:

$$CO(g) + * \xleftrightarrow{K_{CO}} *CO$$

$$K_{CO} = \frac{[*CO]}{P_{CO}[*]} \tag{73}$$

Because *CO is the only major adsorbate, the concentration of all sites $[L]$ can be expressed as:

$$[L] = [*CO] + [*] \tag{74}$$

From equations (73) and (74), we can obtain:

$$[*CO] = \frac{K_{CO}P_{CO}}{1 + K_{CO}P_{CO}}[L] \tag{75}$$

Combining equations (72) and (75) yields:

$$j_{CH_4} = k_{CO(H)} \exp\left(\frac{\beta\eta F}{RT}\right)\frac{K_{CO}P_{CO}}{1 + K_{CO}P_{CO}}[L] \tag{76}$$

At low P_{CO}, according to equation (75), the $[*CO]$ term approaches $K_{CO}P_{CO}[L]$, the rate expression can be expressed as:

$$j_{CH_4} = k_{CO(H)} \exp\left(\frac{\beta\eta F}{RT}\right) K_{CO} P_{CO}[L] \tag{77}$$

The reaction order with respect to P_{CO} is 1.

At high P_{CO}, the $[*CO]$ term approaches $[L]$, the rate expression can be expressed as:

$$j_{CH_4} = k_{CO(H)} \exp\left(\frac{\beta\eta F}{RT}\right)[L] \tag{78}$$

The reaction order with respect to P_{CO} is 0.

Possibility 2: The RDS is the later proton-coupled electron transfer step (*B.4* mechanism).

RDS: $*CO + H_2O + e^- \xrightarrow{K_{CO(H)}} *CO(H) + OH^-$

$$*CO(H) + H_2O + e^- \xrightarrow[RDS]{k_{CO(H)2}} *CO(H)_2 + OH^-$$

The rate expression can be written as:

$$j_{CH_4} = k_{CO(H)_2}[*CO(H)] \exp\left(\frac{\beta\eta F}{RT}\right) \tag{79}$$

where β is the symmetry factor for the reduction process.

We assume a fast equilibrium for CO adsorption on the surface, where the equilibrium constant is K_{CO}:

$$CO(g) + * \xleftrightarrow{K_{CO}} *CO$$

$$K_{CO} = \frac{[*CO]}{P_{CO}[*]} \tag{80}$$

Because *CO is the only major adsorbate, the concentration of all sites $[L]$ can be expressed as:

$$[L] = [*CO] + [*] \tag{81}$$

From equations (80) and (81), we can obtain:

$$[*CO] = \frac{K_{CO}P_{CO}}{1+K_{CO}P_{CO}}[L] \tag{82}$$

Similarly, the generation of $*CO(H)$ is assumed to be a fast equilibrium, and the rate expression of the generation of $*CO(H)$ can be written as:

$$K_{CO(H)} = \frac{K_W[*CO(H)]}{[*CO][H^+]\exp\left(\frac{\eta F}{RT}\right)} \tag{83}$$

Where K_W represents the ion-product constant of water.

Combining equations (79), (82) and (83) yields:

$$j_{CH_4} = \frac{k_{CO(H)_2} K_{CO(H)}[H^+]}{K_W}\exp\left(\frac{(1+\beta)\eta F}{RT}\right)\frac{K_{CO}P_{CO}}{1+K_{CO}P_{CO}}[L] \tag{84}$$

At low P_{CO}, according to equation (82), the $[*CO]$ term approaches $K_{CO}P_{CO}[L]$, the rate expression can be expressed as:

$$j_{CH_4} = \frac{k_{CO(H)_2} K_{CO(H)}[H^+]}{K_W}\exp\left(\frac{(1+\beta)\eta F}{RT}\right)K_{CO}P_{CO}[L] \tag{85}$$

The reaction order with respect to P_{CO} is 1.

At high P_{CO}, the $[*CO]$ term approaches $[L]$, the rate expression can be expressed as:

$$j_{CH_4} = \frac{k_{CO(H)_2} K_{CO(H)}[H^+]}{K_W}\exp\left(\frac{(1+\beta)\eta F}{RT}\right)[L] \tag{86}$$

The reaction order with respect to P_{CO} is 0.

Possibility 3: The RDS is the later chemical hydrogenation step (*B.6* mechanism).

RDS: $*CO + H_2O + e^- \xrightarrow{K_{CO(H)}} *CO(H) + OH^-$

$$*CO(H) + *H \xrightarrow[RDS]{k_{CO(H)2}} *CO(H)_2 + *$$

The rate expression can be written as:

$$j_{CH_4} = k_{CO(H)_2}[*CO(H)][*H] \tag{87}$$

We assume a fast equilibrium for CO adsorption on the surface, where the equilibrium constant is K_{CO}:

$$CO(g) + * \xleftrightarrow{K_{CO}} *CO$$

$$K_{CO} = \frac{[*CO]}{P_{CO}[*]} \tag{88}$$

Similarly, the reduction of H_2O to produce *H is assumed to be a fast equilibrium. We note that the absolute H_{ads} coverage on Cu likely remains low due to the lack of experimental observation

and relatively low hydrogen binding energy.[1] The H_{ads} coverage keep nearly constant with the change of applied potential:

$$H_2O + e^- + * \xleftrightarrow{K_H} *H + OH^-$$

$$K_H = \frac{K_W[*H]}{[H^+][*]} \tag{89}$$

Where K_w represents the ion-product constant of water.

The concentration of all sites $[L]$ can be expressed as:

$$[L] = [*CO] + [*H] + [*] \tag{90}$$

The coverage of H_{ads} is considered to be small:

$$[L] = [*CO] + [*] \tag{91}$$

From equations (88), (89) and (91), we can obtain:

$$[*CO] = \frac{K_{CO}P_{CO}}{1+K_{CO}P_{CO}}[L] \tag{92}$$

$$[*H] = \frac{K_H[H^+]}{K_W\left(1+K_{CO}P_{CO}\right)}[L] \tag{93}$$

The generation of $*CO(H)$ is assumed to be a fast equilibrium, and the rate expression of the generation of $*CO(H)$ can be written as:

$$K_{CO(H)} = \frac{K_W[*CO(H)]}{[*CO][H^+]\exp\left(\frac{\eta F}{RT}\right)} \tag{94}$$

Combining equations (87), (92), (93) and (94) yields:

$$j_{CH_4} = \frac{k_{CO(H)_2}K_{CO(H)}K_H[H^+]}{K_W}\exp\left(\frac{\eta F}{RT}\right)\frac{K_{CO}P_{CO}}{(1+K_{CO}P_{CO})^2}[L]^2 \tag{95}$$

At low P_{CO}, according to equation (92), the $[*CO]$ term approaches $K_{CO}P_{CO}[L]$, the rate expression can be expressed as:

$$j_{CH_4} = \frac{k_{CO(H)_2}K_{CO(H)}K_H[H^+]}{K_W}\exp\left(\frac{\eta F}{RT}\right)K_{CO}P_{CO}[L]^2 \tag{96}$$

The reaction order with respect to P_{CO} is 1.

At high P_{CO}, the $[*CO]$ term approaches $[L]$, the rate expression can be expressed as:

$$j_{CH_4} = \frac{k_{CO(H)_2} K_{CO(H)} K_H [H^+]}{K_W} \exp\left(\frac{\eta F}{RT}\right) \frac{1}{1+K_{CO} P_{CO}} [L]^2 \tag{97}$$

The reaction order with respect to P_{CO} is -1.

Supplementary Note 2: Calculations of local pH during CORR

Calculations of local pH in the buffer solutions at electrolyte pH 7.2 and 9.0

A typical method reported by Hori *et al.*[2] was used to estimate the electrolyte pH at electrode surface in this work. The local pH in the buffer solutions at electrolyte pH 7.2 and 9.0 can be calculated by the following equation:

$$pH = pK_a + \log\left(\frac{C_{2b} + \frac{ir}{FD_2}}{C_{1b} - \frac{ir}{FD_1}}\right) \tag{98}$$

Where K_a represent the equilibrium constant of eq. 1 and eq. 3 at electrolyte pH 7.2 and 9.0 respectively. C_{1b} and C_{2b} are the concentrations of $H_2PO_4^-$ and HPO_4^{2-} at electrolyte pH 7.2 and HCO_3^- and CO_3^{2-} at electrolyte pH 9.0. *D* represent their corresponding diffusion coefficients obtained from *CRC Handbook of Chemistry and Physics 95th*. *F* is the Faraday's constant (96485 C mol^{-1}). *i* is the current density during CORR. *r* is the boundary layer thickness, estimated to be ~1 um based on the reaction-diffusion model reported in previous studies.[3-5]

The pK_a value of specific chemical equations and diffusion coefficients of species are obtained from *CRC Handbook of Chemistry and Physics 95th*.

	Chemical equation	*pKa* at 25 °C
1	$H_2PO_4^- = H^+ + HPO_4^{2-}$	7.21
2	$HPO_4^{2-} = H^+ + PO_4^{3-}$	12.32
3	$HCO_3^- = H^+ + CO_3^{2-}$	10.33

Species	D / (10^{-9} m^2 s^{-1})
OH^-	5.273
CO_3^{2-}	0.923
HCO_3^-	1.185
PO_4^{3-}	0.824
HPO_4^{2-}	0.759
$H_2PO_4^-$	0.959

Calculations of local pH in the buffer solutions at electrolyte pH 11.3, 12.9 and 13.9

OH^- produced at electrode surface will not be neutralized by any species at these alkaline electrolytes. Therefore, the local pH can be calculated by the following equation:

$$pH = 14 + \log\left([OH^-_{\text{formation}}] + [OH^-_{bulk}]\right) \tag{99}$$

Where $[OH^-_{formation}]$ represents the increased concentration of OH^- during the reaction at the electrode surface and $[OH^-_{bulk}]$ represents the concentration of OH^- in the bulk electrolyte. The concentration of OH^- as a function of the distance from the electrode surface can be given by the steady-state governing equation as shown below:

$$D_{OH^-} \frac{d[OH^-]}{dx} = -OH^-_{formation} \tag{100}$$

Where $OH^-_{formation}$ represents the rate of OH^- formation at cathode surface. x represents the distance from electrode surface. D represents the diffusion coefficient of OH^- obtained from *CRC Handbook of Chemistry and Physics 95th*. According to equation (100), the equation (99) can be expressed as:

$$pH = 14 + \log\left(\frac{ri}{D_{OH^-} F} + [OH^-_{bulk}]\right) \tag{101}$$

As detailed in calculations in Supplementary Note 2, the influence of local pH rise is insignificant based on our analysis due to the concentrated buffer employed in near neutral electrolytes and the thin boundary layer of our gas diffusion type electrodes (Supplementary Fig. 3).

Supplementary References

(1) Sheng, W.; Myint, M.; Chen, J. G.; Yan, Y. Correlating the Hydrogen Evolution Reaction Activity in Alkaline Electrolytes with the Hydrogen Binding Energy on Monometallic Surfaces. *Energy Environ. Sci.*, **6**, 1509 (2013).
(2) Hori, Y.; Murata, A.; Takahashi, R. Formation of Hydrocarbons in the Electrochemical Reduction of Carbon-Dioxide at a Copper Electrode in Aqueous-Solution. *J. Chem. Soc., Faraday Trans. 1*, **85**, 2309-2326 (1989).
(3) Burdyny, T.; Smith, W. A. CO_2 Reduction on Gas-Diffusion Electrodes and Why Catalytic Performance Must Be Assessed at Commercially-Relevant Conditions. *Energy Environ. Sci.*, **12**, 1442-1453 (2019).
(4) Weng, L. C.; Bell, A. T.; Weber, A. Z. Modeling Gas-Diffusion Electrodes for CO_2 Reduction. *Phys. Chem. Chem. Phys.*, **20**, 16973-16984 (2018).
(5) Chen, Y.; Lewis, N. S.; Xiang, C. Modeling the Performance of A Flow-Through Gas Diffusion Electrode for Electrochemical Reduction of CO or CO_2. *J. Electrochem. Soc.*, **167**, 114503 (2020).
